# Performance Drift in a Nationally Deployed Population Health Risk Algorithm in the US Veterans Health Administration

**DOI:** 10.1001/jamahealthforum.2025.2717

**Published:** 2025-08-15

**Authors:** Likhitha Kolla, Kristin Linn, Amol S. Navathe, Craig Kreisler, Christopher B. Roberts, Sae-Hwan Park, Harvineet Singh, Jean Feng, Jinbo Chen, Ravi B. Parikh

**Affiliations:** 1 Perelman School of Medicine, University of Pennsylvania, Philadelphia; 2 US Department of Veterans Affairs, Center for Health Equity Research and Promotion, Corporal Michael J. Crescenz VA Medical Center, Philadelphia, Pennsylvania; 3 Office of Quality and Patient Safety, Veterans Health Administration, Washington, DC; 4 The Parity Center, Perelman School of Medicine, University of Pennsylvania, Philadelphia; 5 Department of Epidemiology and Biostatistics, University of California, San Francisco; 6 Emory University School of Medicine, Emory University, Atlanta, Georgia; 7 Winship Cancer Institute, Atlanta, Georgia

## Abstract

**Importance:**

Clinical risk algorithms inform clinical decision support and system-level quality metrics. However, algorithm performance can drift over time and possibly promote misinformed decision-making and resource allocation. The Veterans Health Administration (VA) Care Assessment Needs (CAN) algorithm is a nationally deployed population risk algorithm used to predict risk of 90-day hospitalization and/or mortality and to allocate resources for more than 5 million veterans annually. However, drift affecting the VA CAN has not been assessed.

**Objective:**

To evaluate the impact of drift in the VA CAN algorithm and the extent, mechanisms, and clinical consequences of performance changes.

**Design, Setting, and Participants:**

This was a retrospective cohort study using electronic health records (EHRs) and administrative data from the VA Corporate Data Warehouse, which contains observations from more than 5 million veterans per study year. The data comprised 27 787 152 observations among 7 215 711 unique veterans receiving VA primary care from 2016 to 2021. Data analysis was performed from January 2023 and December 2024.

**Main Outcomes and Measures:**

Two primary outcomes were change in model performance (true positive rate [TPR], false positive rate [FPR], positive predictive value [PPV], negative predictive value [NPV], F1 score, and accuracy); and a national quality metric (% of veterans with CAN ≥90th percentile with a palliative care visit).

**Results:**

The study population included 7 215 711 eligible veterans, with a mean (SD) age of 62.1 (16.5); 91.2% were male and 18.2% were Black, 6.6% Hispanic, and 76.2% White individuals. From 2016 to 2021, PPV decreased by 4.0% (95% CI, −2.8% to −5.1%); F1 score decreased by 4.6% (95% CI, −6.1% to 3.0%); NPV increased by 0.43% (95% CI, 0.30% to 0.57%); and FPR increased by 0.34% (95% CI, 0.10% to 0.58%), which corresponds with 18 288 increased false positive results. TPR and accuracy remained stable. The 90-day hospitalization and/or death rates decreased from 3.8% in 2017 to 3.0% in 2021. Covariate shifts were observed in 19 covariates, with demographic characteristics, health care utilization, and laboratory covariates exhibiting the largest shifts. The palliative care quality metric was 2.9% (95% CI, 2.8% to 2.9%) in 2018, 2.6% (95% CI, 2.6% to 2.7%) in 2019, and 2.8% (95% CI, 2.7% to 2.8%) in 2020, with FPRs among metric-eligible veterans increasing from 81.6% (95% CI, 81.5% to 81.7%) in 2018 to 85.7% (95% CI, 85.6% to 85.8%) in 2020.

**Conclusions and Relevance:**

This cohort study found that CAN algorithm performance declined from 2016 to 2021 due to shifts in outcome prevalence and distributions of health care utilization and demographic covariates. Close surveillance of clinical risk algorithms and quality metrics derived from algorithm-generated risk scores could mitigate suboptimal resource allocation or decision-making.

## Key Points

**Question:**

What are the extent, mechanisms, and clinical consequences of performance changes in the Care Assessment Needs (CAN) algorithm, a nationally deployed population health-risk algorithm used by the Veterans Health Administration?

**Findings:**

In this retrospective cohort study of 27 787 152 observations among 7 215 711 unique veterans, with more than 5 million observations from this patient pool recorded annually, CAN algorithm positive predictive value decreased by 4.00% and false positive rate increased by 0.34% from 2016 to 2021, corresponding to 18 288 increased false positives. Demographic, health care utilization, and laboratory variables were significant contributors to drift.

**Meaning:**

These findings suggest that clinical risk algorithms such as the CAN algorithm are susceptible to performance drift, particularly during shifts during time periods associated with the COVID-19 pandemic, contributing to decreased reliability of quality metrics; therefore, regular monitoring and adaptation of algorithms should be used to ensure continued clinical utility.

## Teaser

This cohort study evaluates magnitude, mechanisms, and impact of drift on the performance of the Veterans Health Administration’s Care Assessment Needs algorithm.

## Introduction

Clinical risk algorithms using routinely collected health care data (eg, claims, electronic health records) as inputs may be utilized to predict adverse outcomes and applied to clinical decision-making support, risk stratification, and risk adjustment.^1,2,3^ However, these algorithms may exhibit what is termed *performance drift*, a deterioration in algorithm performance over time.^4,5^ Performance drift may support misinformed clinical decision-making and/or inefficient allocation of health care resources. For example, early in the COVID-19 pandemic, a widely implemented sepsis prediction algorithm generated increased alerts that likely were responsible for care disruptions and/or inefficient use of rapid response systems.^6^ As recognition of drift grows, the US Food and Drug Administration^7^ and The White House Blueprint for an AI Bill of Rights^8^ have advocated for monitoring frameworks to track and understand contributors to drift, provide mechanism-guided mitigation strategies to avoid premature decommissioning, and prevent patient safety impacts.

Drift can arise from differences in hospital settings, time periods, or patient demographic characteristics between when an algorithm is developed and when it is used.^4^ Two primary causes of drift include shifts in patient characteristics and changes in outcome prevalence.^4,9^ For example, the previously mentioned sepsis algorithm may have been less reliable during the COVID-19 quarantine period due to increased acuity of patient case mix and greater prevalence of in-hospital mortality.^6^ Although previous studies have quantified performance changes in clinical risk algorithms,^5,6,10,11,12^ few have evaluated how drift affects clinical decisions and outcomes in the routine community setting.^13,14^ Additionally, there are a lack of methods to detect when and why performance changes occur, which limits efforts to develop effective monitoring and mitigation strategies.^7,8,15,16,17,18^

To this end, we sought to study the extent, associated factors, and clinical impact associated with performance drift by leveraging the Veterans Health Administration (VA) Care Assessment Needs (CAN) algorithm, a nationally deployed tool that generates a risk score representing the predicted 90-day likelihood of a combined hospitalization and/or mortality outcome for more than 5 million veterans accessing VA primary care services. We studied the CAN algorithm for 2 reasons: it informs population-level case management strategies and quality reporting among a diverse VA population; and the dataset contains longitudinal information on inputs and outcomes, enabling us to track the magnitude of drift over time, which variables are associated with drift, and the clinical impact of drift.

## Methods

This study was approved and informed consent requirement was waived by the institutional review board at the Corporal Michael J. Crescenz VA Medical Center (Philadelphia, Pennsylvania) owing to use of retrospective summary-level data. We followed the Strengthening the Reporting of Observational Studies in Epidemiology (STROBE) reporting guideline.

### Study Design, Population, and Setting

This retrospective cohort study assessed electronic health records (EHRs) and administrative data from the VA Corporate Data Warehouse between 2016 to 2021, a national repository integrating data from multiple VA clinical and administrative systems. The dataset includes records for more than 5 million veterans enrolled in VA primary care services annually. Patient demographic characteristics (including race and ethnicity), medical history, prescribed medications, vital signs, laboratory results, health care utilization, and final risk scores calculated per calendar year (CY) were extracted from VA EHRs (VA VitalStatus file, Corporate Data Warehouse, and Observational Medical Outcomes Partnership variables). The more recent score available in December of each CY was analyzed. The 90-day combined outcomes measured in March of the following CY were studied.

### VA CAN Score

We studied the VA CAN risk algorithm, version 2.5, which estimates the likelihood of a 90-day combined hospitalization and/or mortality outcome. The CAN algorithm was trained on 2016 VA EHR data and deployed in January 2019. We focused on the 90-day combined score because risk classifications derived from it have been used by the VA to inform quality metrics, including the one we studied. In practice, risk scores are generated by the algorithm on a weekly basis for veterans enrolled in primary care services but are not calculated for those hospitalized at the time of the risk score calculation. In this study, all veterans enrolled in primary care services were included, and scores were recalculated for all veterans using the original logistic regression algorithm coefficients (eTable 1 in Supplement 1). No veterans were excluded from the study. Raw probabilities were transformed into percentile rankings between 0 to 99 to generate the CAN score.

### Study Period

Algorithm performance can deteriorate due to subtle, long-term changes in practice patterns, and/or sudden shocks to health care operations. We separated these 2 causes by measuring performance drift and mechanisms over 3 time periods: 2016 to 2018 (2017-2019), 2019 to 2020 (2020-2021), and 2016 to 2020 (2017-2021). Ranges are notated as year(s) of risk score (year[s] of outcome measurement). The 2016 to 2018 (2017-2019) time period captures changes in covariate distributions and algorithm performance before the COVID-19 pandemic. The 2019 to 2020 (2020-2021) drift period isolates changes in health care practice patterns during the initial period of the COVID-19 pandemic. The 2016 to 2020 (2017-2021) drift time period serves as a composite period, including the impact of both prepandemic and pandemic changes on algorithm performance.

### Quantifying Performance Drift

Drift was measured using 6 metrics of both statistical and operational relevance: true positive rate (TPR), false positive rate (FPR), positive predictive value (PPV), negative predictive value (NPV), F1 score (harmonic mean of TPR and PPV), and accuracy (eAppendix 1 in Supplement 1 provides definitions and further details). In relation to existing clinical literature, TPRs are equivalent to sensitivity, and FPRs are equivalent to 1 specificity. Drift was measured as the absolute percentage change in a performance metric value from the first (baseline) and final year of each period. For example, in the 2016 to 2018 (2017-2019) time period, change was calculated as a difference between the metric in 2016 (2017) and 2020 (2021). In the 2019 to 2020 (2020-2021) time period, 2019 (2020) was used as the baseline metric. The primary measure of drift was at the 90th risk percentile threshold used in several VA operations, with calculations at the 50th, 70th, 95th, 98th, and 99th thresholds in sensitivity analyses. As a secondary analysis, the number of individuals affected by drift was measured by multiplying the absolute change in TPR and FPR by the number of cases and controls, respectively, in the final year of each given time period. As a supplementary analysis, model calibration across 10 risk deciles and area under the curve were calculated for each study year to measure individual-level model metrics.

### Identifying Drift Mechanisms

Drift can occur due to changes in covariate and/or outcome distributions (eAppendix 2 in Supplement 1). Change in outcome distribution was calculated as the absolute change in prevalence of the combined outcome. Covariate shift, change in covariate (ie, input) distributions, was calculated as the standardized mean difference (SMD) in the prevalence of each CAN covariate between the first and final year of each time period. Covariates were divided into 5 categories: demographic, diagnostic, laboratory and vital signs, pharmacy, and utilization data. SMDs were mapped against the original algorithm odds ratios (ORs) to jointly assess covariate shift and original effect size (eAppendix 3 in Supplement 1). Covariates with large shifts (SMD, ≥0.1 or <−0.1 SMD) and/or greatest predictive effects (OR, ≥1.5 or <0.5) were considered to be associated with drift. To understand the impact of these factors on performance drift, the VA CAN algorithm was retrained on 2016 VA EHRs, excluding the identified associated covariates, and the extent of drift was recalculated. Algorithm retraining that excluded factors was compared against algorithm retraining with 2018 and 2019 EHR data with the full covariate set.

### Determining Clinical Impact of Drift

Performance drift affects the reliability of system-level quality metrics informed by clinical risk classification, specifically the number of high-risk patients flagged based on their generated risk score (eTable 2 in Supplement 1). Drift is associated with reduced reliability when the high-risk group no longer represents true high-risk patients. This study assessed the impact of performance drift on a quality metric adapted from the VA, used by several VA offices (eg, the Office of Geriatric and Extended Care, Veterans Experience Center) to track high-risk veterans receiving palliative care. We calculated the following end points of clinical impact: (1) quality metric, which we adapted from the published VA quality metric was the proportion of high-risk veterans (CAN ≥90) who received a palliative care visit; (2) the proportion of false positives among high-risk veterans; and (3) the proportion of false positives among high-risk patients who received palliative care. The latter 2 metrics reflected reduced reliability of the high-risk denominator used in the quality metric. The outcome of palliative care was defined as the first palliative care visit in a CY, identified via VA-defined procedural and EHR identifiers (eTable 3 in Supplement 1). End points were calculated across the 2018, 2019, and 2020 CYs that covered observations before and during the COVID-19 pandemic.

### Statistical Analyses

Bootstrap resampling with replacement at the individual level, which preserves within-individual correlation across repeated longitudinal measures (n = 1 000 000 participants per sample due to computational considerations for 2000 samples) was used to generate nonparametric CIs for performance metrics and drift measures.^17^ The 95% CIs were generated by taking values at the 2.5th and 97.5th quantile of the bootstrap distribution. Because the sampled bootstrap population was smaller than the patient pool, the calculated CIs were conservatively expected to be larger than true CIs. VA CAN algorithm coefficients were considered fixed, and the algorithm was not retrained in each bootstrap sample. CIs for classification rates, outcome prevalence, and clinical impact measures were calculated using the exact binomial method. Data analyses and preprocessing were performed from January 2023 and December 2024 on the Veterans Informatics and Computing Infrastructure remote server with SAS, enterprise 8.3 (SAS Institute Inc). Visualizations were generated with R, version 4.3.3 (R Foundation for Statistical Computing).

## Results

We studied 27 787 152 observations from across 7 215 711 unique veterans from 2016 to 2021, with approximately 5 million observations recorded annually from this unique patient population (Table 1). Across the study years, 2016 (2017) to 2020 (2021), sample demographic characteristics and comorbidities were consistent: mean (SD) age, 62.1 (16.5); 8.0% to 9.6% female and 92.0% to 90.4% male; 17.6% to 18.8% Black, 6.3% to 7.1% Hispanic or Latino, and 77.2% to 75.1% White individuals; 62.6% to 64.1% urban-dwelling. Mean Elixhauser comorbidity count ranged from 2.13 to 2.21 across years; notable prevalent comorbidities included dementia (3.9%-4.4%), congestive heart failure (4.7%-5.1%), and metastatic cancer (7.7%-8.5%).

**Table 1.** Descriptive Statistics of Study Population, Stratified by Study Year

Covariate	Participants, %^a^				
	2016 (2017)	2017 (2018)	2018 (2019)	2019 (2020)	2020 (2021)
Total participants, No.	5 583 397	5 600 275	5 519 660	5 594 791	5 489 029
Female	8.0	8.4	8.7	9.1	9.6
Male	92.0	91.6	91.3	90.9	90.4
Age, mean (SD), y	62.1 (16.4)	62.1 (16.5)	62.1 (16.5)	62.1 (16.6)	62.1 (16.6)
Race^b^					
Black	17.6	17.9	18.2	18.5	18.8
White	77.2	76.7	76.2	75.6	75.1
Other	3.1	3.1	3.2	3.3	3.4
Missing data	2.2	2.3	2.4	2.7	2.8
Ethnicity					
Hispanic or Latino	6.3	6.4	6.6	6.9	7.1
Not Hispanic or Latino	90.8	90.7	90.6	90.3	89.7
Unknown	2.9	2.8	2.8	2.9	3.3
Geographic location^c^					
Urban	62.6	63.0	63.6	64.2	64.1
Rural	35.0	34.9	34.8	34.7	34.7
Missing	2.5	2.1	1.6	1.1	1.2
Medical comorbidities					
Elixhauser Comorbidity count, mean (SD)	2.1 (2.1)	2.2 (2.1)	2.3 (2.2)	2.3 (2.2)	2.2 (2.2)
Dementia	4.2	4.3	4.4	4.4	3.9
Congestive heart failure	4.7	4.9	5.0	5.1	4.8
Metastatic cancer	7.7	7.9	8.4	8.5	7.9
Prediction score^d^					
Mean (SD)	3.7 × 10^−2^ (6.3 × 10^−2^)	3.8 × 10^−2^ (6.4 × 10^−2^)	3.9 × 10^−2^ (6.4 × 10^−2^)	3.9 × 10^−2^ (6.4 × 10^−2^)	3.7 × 10^−2^ (5.9 × 10^−2^)
Median (IQR: Q3-Q1)	1.7 × 10^−2^ (2.4 × 10^−2^)	1.7 × 10^−2^ (2.5 × 10^−2^)	1.8 × 10^−2^ (2.6 × 10^−2^)	1.8 × 10^−2^ (2.6 × 10^−2^)	1.9 × 10^−2^ (2.6 × 10^−2^)
90-d Hospitalization and/or mortality	3.8	3.9	3.9	3.1	3.0

Abbreviations: CAN, Care Assessment Needs algorithm; VA, US Department of Veterans Affairs.

^a^ Year is notated as year of risk score calculation (year of outcome measurement).

^b^ Race and ethnicity data, including the category “other,” were derived from VA VitalStatus file, Corporate Data Warehouse, and Observational Medical Outcomes Partnership variables.

^c^ Variable from the Corporate Data Warehouse (GISURH) indicating geographic location and connected to a patient address. For each patient record, the most recent address record starting before or at the CAN risk date was used. Rural is a composite metric to represent veterans who reside in rural and highly rural locations, as defined by the VA.

^d^ CAN scores are updated on a weekly basis, but the last algorithm run for each calendar year was analyzed for this study. Prediction scores reflect the raw probability of the 90-day outcome and are reported between 0 and 1.

**Figure 1.**
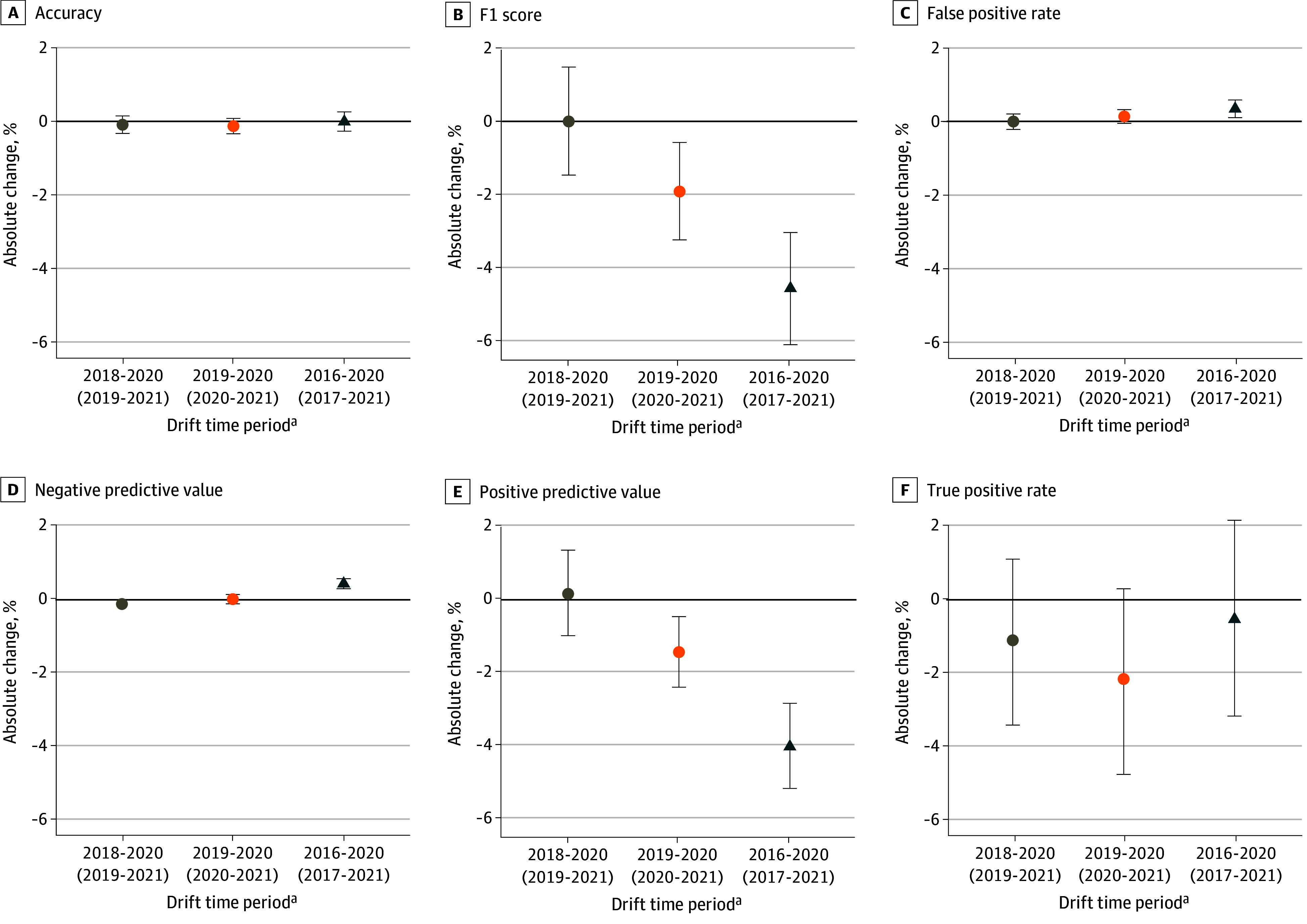
Performance Drift in Veterans Affairs (VA) Care Assessment Needs (CAN) Algorithm Across Time Period at the 90th Percentile Risk Threshold, by Classification Metric ^a^Performance drift across 6 metrics (ie, accuracy F1 score, false positive rate, negative predictive value, positive predictive value, and true positive rate), with metrics calculated at the 90th percentile risk threshold. Performance drift was measured as the absolute percentage change in each performance metric (y-axis) from baseline metrics and was calculated for 3 different drift time periods. Baseline metrics were derived from 2016, 2019, and 2016 for the 3 time periods, respectively. Year is notated as year of risk score (year of outcome measurement). The solid horizontal line at y = 0 represents no change in the metric across the time period. Whiskers indicate that 95% CIs of each performance drift point estimate. Sensitivity analyses, performance metrics by year, and values of drift, definitions of performance metrics, and description on how 95% bootstrapped CIs were obtained are detailed in Supplement 1. F1 score is the harmonic mean of true positive rate and positive predictive value.

At the 90th percentile, the greatest performance drift was observed in the 2016 to 2020 (2017-2021) and 2019 to 2020 (2020-2021) time periods (Figure 1; eFigure 1 and eTables 4-5 in Supplement 1). In the 2016 to 2020 (2017-2021) period, F1 scores decreased from 26.6% (95% CI, 25.5% to 27.6%) to 22.0% (95% CI, 21.1% to 23.0%) (absolute change, −4.6%; 95% CI, −6.1% to −3.0%); NPV increased from 97.8% (95% CI, 97.7% to 97.9%) to 98.3% (95% CI, 98.2% to 98.4%) (absolute change, 0.4%; 95% CI, 0.3% to 0.6%); PPV decreased from 18.3% (95% CI, 17.5% to 19.1%) to 14.3% (95% CI, 13.6% to 15.0%) (absolute change, −4.0%; 95% CI, −5.1% to −2.8%); and FPR increased from 8.5% (95% CI, 8.3% to 8.7%) to 8.8% (95% CI, 8.7% to 9.0%) (absolute change, 0.4%; 95% CI, 0.1% to 0.6%). Notable drift in TPR and accuracy was not observed between 2016 to 2020 (2017-2021). In the 2019 to 2020 (2020-2021) time period, F1 scores decreased from 23.9% (95% CI, 22.9% to 24.9%) to 22.0% (95% CI, 21.1% to 23.0%) (absolute change, −1.9%; 95% CI, −3.2% to −0.6%); PPV decreased from 15.7% (95% CI, 14.9% to 16.5%) to 14.3% (95% CI, 13.6% to 15.0%) (absolute change, 1.4%; 95% CI, 0.5% to 2.4%); and FPR increased from 8.7% (95% CI, 8.5% to 8.9%) to 8.8% (95% CI, 8.7% to 9.0%) (absolute change, 0.1%; 95% CI, −0.1% to 0.3%). These changes corresponded to estimated increases of 18 288 and 7122 false positives in the 2016 to 2020 (2017-2021) and 2019 to 2020 (2020-2021) time periods, respectively (eFigure 2 in Supplement 1). In the 2019 to 2020 (2020-2021) time period, TPR decreased from 50.2% (95% CI, 48.2% to 51.9%) to 48.0% (95% CI, 46.2% to 50.0%) (absolute change, −2.2% [95% CI, −4.9% to 0.3%]), corresponding to an estimated decrease of 3534 veterans with 90-day hospitalization or mortality who were identified as high risk by the algorithm-generated risk score (eFigure 3 in Supplement 1). We did not observe significant drift in the 2016 to 2018 (2017-2019) time period in any performance metric. Trends in performance drift were consistent at the 50th, 70th, 95th, 98th, and 99th risk percentile thresholds (eFigure 1 in Supplement 1), with smaller magnitudes of drift at the highest risk thresholds.

Table 2 displays algorithm classification rates at the 90th risk percentile threshold and outcome prevalence across study years. During the study period, the proportion of individuals classified as true positives decreased from 1.8% to 1.4%. The proportion of true negatives remained stable across years, from 88.1% to 88.5%, while false positives increased from 8.2% to 8.6%. The overall outcome prevalence decreased from 3.8% in 2017 to 3.0% in 2021. Trends in classification rates were consistent across risk percentile thresholds (eTable 6 in Supplement 1).

**Table 2.** Algorithm Classification Rates and Outcome Prevalence, By Year

Year^a^	% (95% CI)				
	True positives	True negatives	False positives	False negatives	Outcome prevalence
2016 (2017)	1.82 (1.81-1.83)	88.05 (88.03-88.08)	8.17 (8.14-8.19)	1.94 (1.92-1.95)	3.76 (3.75-3.78)
2017 (2018)	1.87 (1.85-1.87)	87.98 (87.95-88.00)	8.13 (8.10-8.15)	2.01 (2.00-2.03)	3.88 (3.86-3.90)
2018 (2019)	1.84 (1.83-1.85)	87.95 (87.92-87.97)	8.15 (8.13-8.17)	2.04 (2.03-2.05)	3.89 (3.87-3.90)
2019 (2020)	1.57 (1.56-1.58)	88.43 (88.41-88.46)	8.42 (8.40-8.45)	1.56 (1.55-1.57)	3.13 (3.11-3.14)
2020 (2021)	1.42 (1.41-1.43)	88.45 (88.42-88.47)	8.57 (8.54-8.59)	1.54 (1.53-1.55)	2.97 (2.96-2.98)

^a^ Year is notated as year of risk score calculation (year of outcome measurement).

Changes in algorithm classification rates from 2016 to 2021 at the 90th percentile threshold. The last column includes the outcome prevalences across all study years. Sensitivity analyses of rates at 50th, 70th, 95th, 98th, and 99th percentile risk threshold reported in eTable 6 in Supplement 1.

**Figure 2.**
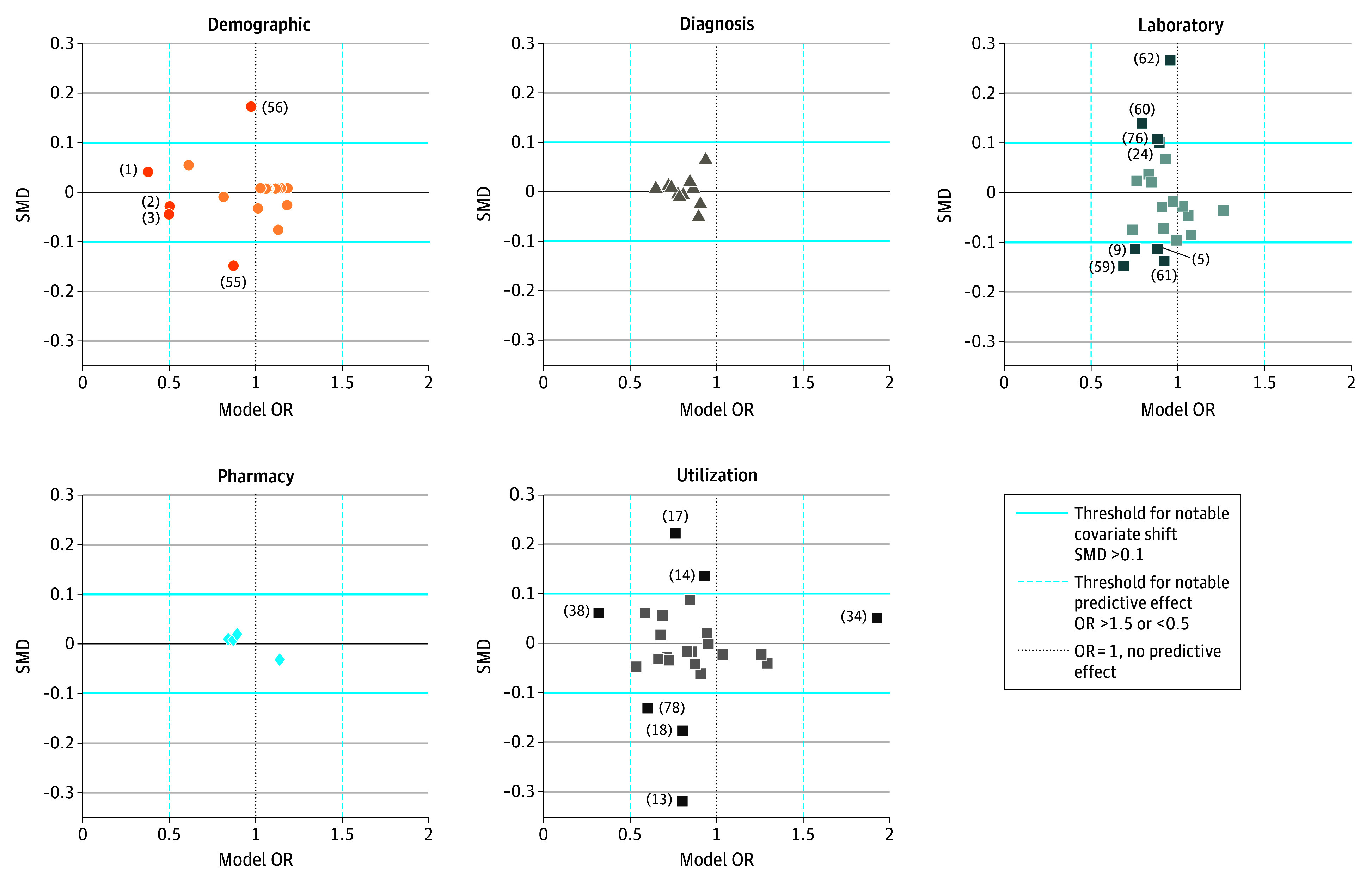
Covariate Shift in the VA Care Assessment Needs (CAN) Algorithm From 2016 to 2020 (2017 to 2021), by Covariate Category and Variable Identification (ID)^a^ ^a^Key for variable IDs (unless otherwise stated, all values are the most recent within the preceding 1 year): (1) age <55 vs ≥ 85 years; (2) age 55-64 vs ≥ 85 years; (3) age 65-74 vs ≥ 85 years; (5) albumin, >3.4 g/dL vs no result; (9) blood urea nitrogen, 0-16 mg/dL vs no result; (10) blood urea nitrogen, 17-25 mg/dL vs no result; (13) telephone consultation CPT code for 21-30 mins in prior 2 years, 0 vs >1; (17) established office visit CPT code in prior 90 days, 0 vs >4 visits; (18) established office visit CPT code in prior 90 days, 1-4 vs >4 visits; (34) hospital stays/bed days of care, level 0 vs 4; (38) hospital admission, 0 vs >2 admissions; (55) veteran priority, level 0 vs 5; (56) veteran priority, level 1 vs 5; (59) pulse, <60 vs ≥90 beats/min; (60) pulse, 60 to <90 or unknown vs ≥90 beats/min; (61) respiration, <18 vs ≥20 breaths/min; (62) respiration, 18 to <20 or unknown vs ≥20 breaths/min; (75) BP, <110 vs ≥160 mmHg; (76) BP, 110 to <140 or unknown vs ≥160 mmHg. Covariates outside of the horizontal solid lines represent thresholds for significant covariate shift and those outside the vertical dotted lines are potential factors associated with drift. Main factors are related to care priority status; hospital utilization patterns (eg, office visits vs telehealth visits, hospital admissions, hospital stays and bed days of care, and outpatient visits); and laboratory markers (eg, albumin, blood urea nitrogen, BP, and respiration and pulse vitals). Original algorithm ORs and covariate shifts (2019-2020 and 2016-2018) are available in the eTables 1 and 7 and eFigures 4 and 5 in Supplement 1, respectively. BP indicates systolic blood pressure; CPT, Current Procedural Terminology code; OR, odds ratio; SMD, standardized mean difference; VA, US Department of Veterans Affairs.

Figure 2 presents covariate shift in the 2016 to 2020 (2017-2021) time period for each CAN covariate. Pronounced distributional shifts were observed in 19 covariates belonging to the following categories: demographic characteristics (ie, veteran priority status), utilization (ie, outpatient visits, telehealth use, office visits, hospital admissions, hospital stays, and bed-days of care), and laboratory and vital signs (ie, albumin, blood urea nitrogen, blood pressure, pulse, and respiration measurements) (eTable 7 in Supplement 1). Covariates belonging to diagnostic and pharmacy categories remained close to their baseline distribution. Most covariate shifts were observed in the 2019 to 2020 (2020-2021) period (eFigure 4 in Supplement 1); notable shifts in covariate distributions were not observed in the 2016 to 2018 (2017-2019) period (eFigure 5 in Supplement 1). Removing the factors both in total and by variable type did not alleviate the extent of drift among any performance metric (eTable 8 in Supplement 1).

Table 3 represents changes in the magnitude and composition of a standard VA quality metric, defined as the proportion of patients flagged as high risk who received a palliative care visit. The metric fluctuated from 2.9% (95% CI, 2.8% to 2.9%) in 2018 to 2.6% (95% CI, 2.6 to 2.7) in 2019 to 2.8% (95% CI, 2.7 to 2.8) in 2020. The percentage of false positives increased from 81.6% (95% CI, 81.5% to 81.7%) to 85.7% (95% CI, 85.6% to 85.8%). Among high-risk patients who received a palliative care visit, the percentage of false positives increased from 61.2% (95% CI, 60.5% to 62.0%) in 2018 to 69.1% (95% CI, 68.3% to 69.8%) in 2020. Similar trends were observed at the operationally relevant 98th and 99th risk percentile thresholds (eTable 9 in Supplement 1).

**Table 3.** Impact of Performance Drift on Quality Metric Reliability Across 3 Fiscal Years, 2018 to 2020

Year^a^	% (95% CI)		
	High-risk patients who received palliative care (quality metric)	FPR in high-risk veterans	FPR in high-risk veterans who received palliative care
2018 (2019)	2.89 (2.84-2.93)	81.55 (81.45-81.65)	61.21 (60.45-61.96)
2019 (2020)	2.61 (2.57-2.65)	84.29 (84.20-84.39)	64.72 (63.95-65.50)
2020 (2021)	2.76 (2.71-2.80)	85.72 (85.63-85.81)	69.06 (68.32-69.80)

Abbreviations: FPR, false positive rate; VA, US Department of Veterans Affairs.

^a^ Year is notated as year of risk score calculation (year of outcome measurement).

VA quality metric values and FPR among those flagged as high risk at the 90th percentile risk threshold and in the high-risk group who received a palliative care visit. Results for other operationally relevant thresholds (ie, 98th and 99th risk) are in eTable 9 in Supplement 1. Percentage of high-risk patients who received palliative care represents the VA-adapted quality metric and measures the proportion of the high-risk veterans who received a palliative care visit. FPR among high-risk veterans is numerically equivalent to 1-positive predictive value and refers to the proportion of individuals identified as high-risk by the Care Assessment Needs algorithm (ie, score ≥90th percentile) who did not experience the 90-day combined outcome (hospitalization and/or mortality). FPR among high-risk veterans who received palliative care reflects the proportion of veterans who were flagged as both high-risk and received a palliative care visit but did not experience the 90-day outcome.

## Discussion

We studied the extent, mechanisms, and clinical impact of performance drift in the VA CAN algorithm, a nationally implemented clinical risk algorithm. Between 2016 and 2021, the CAN algorithm exhibited worse performance in clinically and operationally relevant metrics, including TPR, FPR, PPV, and F1 score. Pronounced changes occurred during the COVID-19 pandemic, coinciding with sudden shifts in hospitalization and mortality cases, in health care and laboratory utilization, and in patient case mix. Algorithmic instability can undermine the validity of downstream hospitality quality metrics. In our study, we observed shifts in a palliative care quality metric used for quality reporting by the VA, which relies on accurately identifying patients at high-risk for hospitalization and/or mortality outcomes.

We identified 2 possible mechanisms of performance deterioration. First, changes in how often the outcome occurred and shifts in key patient characteristics used by the algorithm contributed to drift. Specifically, a drop in the prevalence of the combined hospitalization and/or mortality cases was associated with fewer high-risk patients being correctly identified (PPV decline) and a greater number of low-risk patients incorrectly flagged by the algorithm-generated risk score (FPR increase).^9,19,20^ Outcome prevalence can vary for several reasons, including changes in public health risks (eg, increased mortality due to emerging diseases or reduced hospitalizations due to quarantine measures), improvements in data quality, updates in diagnostic criteria, and shifts in patient case mix.^4,15^ Second, algorithm inputs that have a large influence on risk calculation and fluctuate in frequency as health care environments change could have driven drift.^15^ In our study, inputs that shifted had low individual influence but collectively had a large influence on CAN scores. Most of these inputs were related to changes in health care and laboratory utilization, including factors such as hospital admissions, bed days of care, in person clinic visits, telehealth, and vital (eg, albumin, blood urea nitrogen) measurements. Changes in these inputs likely reflect COVID-19 pandemic−driven shifts in health care access and delivery. Regular monitoring of the magnitude and frequency of shifts in key patient characteristics, utilization patterns, and outcome prevalence can guide mitigation strategies. For example, small but frequent shifts would benefit from recalibration (adjustment of algorithm outputs without modifying underlying algorithm structure or input weights), while large sustained shifts—eg, the expanded use of telehealth after quarantine—may require algorithm retraining (updating algorithm structure and/or weights of inputs using new data). Various recalibration approaches exist, including Platt scaling and isotonic regression, which apply monotonic transformations that preserve the relative ordering of predicted risk scores.^21^ Because we are working with the CAN score—a percentile ranking of the raw predicted risk scores—recalibrating the raw scores may not be appropriate for adjusting classifications or performance metrics. Additional mitigation strategies that modify the algorithm in dynamic clinical environments include adaptive machine learning approaches, which regularly update the algorithm with new data while preserving historical information; flexible thresholding, which adjust decision boundaries in response to changes in covariate distributions; and feature dropping, which eliminates covariates that have lost predictive value over time. Considerations for selecting the appropriate mitigation strategy include the type, extent, and longevity of drift; computational resources; and clinical impact.^22,23,24,25,26^

Our findings emphasize the need for robust monitoring systems tailored to the clinical context in which algorithms are deployed. Prior studies on drift have focused on broad statistical measures, such as overall accuracy, which lack direct insight into clinical or operational consequences.^5,6,11,27,28^ In contrast, we observed drift in clinically relevant population-level metrics that directly impact care decisions and resource allocation. For example, while we observed stability in algorithm accuracy, we found drift in measures such as TPR and PPV, which impact how often high-risk patients were correctly identified and how accurately flagged patients experienced the predicted outcome. We further found drift in calibration, which assesses concordance between predicted and observed outcomes at the individual level (eTable 10 in Supplement 1). We found that although AUC remained stable, the model is miscalibrated at the highest predicted risk deciles, where clinical decision-making is the most relevant, indicating decay in model performance for individuals at highest risk. Furthermore, because the observed drift was not consistent in direction or magnitude across metrics, understanding the operational use case of a risk algorithm will help prioritize which metric(s) to monitor (eTable 2 in Supplement 1). For example, in a hospitalization-only prediction algorithm, monitoring metrics that help avoid unnecessary interventions caused by false positives, such as PPV and FPR, are essential.^6^ Conversely, for mortality prediction algorithms whose generated risk scores are used to prioritize palliative care visits, metrics that ensure that high-risk patients are correctly identified (eg, TPR) are critical to prevent missed opportunities for timely care.

This study distinguished between gradual and sudden drift periods, which we measured as drift occurring between 2016 to 2018 (2017-2019) and 2019 to 2020 (2020-2021), respectively. Between 2016 and 2019, we observed relatively minor drift in the VA CAN algorithm. However, algorithms implemented in health care settings with more diverse patient populations, minority subgroups within hospital populations, different EHR systems, or rapidly evolving care delivery practices may exhibit greater gradual drift. Monitoring performance recovery in the postpandemic era, when health care operations resumed, could provide insight into how algorithms regain or maintain stability after rapidly changing clinical environments.

There are 4 policy implications of this work. First, similar to the Food and Drug Administration−mandated postmarket surveillance for drugs to ensure ongoing safety and efficacy in clinical settings, clinical risk algorithms should undergo regular performance audits for postmarket recertification. These audits should incorporate internal validation (eg, performance metrics, calibration) and external validation (eg, shifts in patient mix, clinical workflows) to safeguard their reliability in dynamic health care environments. Guidelines from regulatory bodies could set standards for acceptable drift thresholds and recommend timely updates. Second, hospital-level surveillance should send real-time alerts to end users if performance deviations are expected to negatively affect clinical care. Third, institutions should build literacy around performance drift among health care workers to promote responsible use of algorithms and to avoid overreliance.^29^ Fourth, timely drift mitigation should include recalibration of algorithms for small, frequent shifts in performance and/or algorithm retraining for larger, sustained shifts.

### Limitations

This study had limitations worth noting. First, the data were from a single health care system, the VA, limiting the generalizability of our findings. However, the VA is the nation’s largest health care system, serving diverse geographic and demographic populations. Second, we systematically identified changes in algorithm inputs, although these changes among inputs likely occurred in unison. For instance, changes in health care utilization may affect access to laboratory services, altering the distribution of laboratory values. Future work should consider the impact of these multivariate shifts on drift. Third, we did not quantify how time-varying factors in health care, such as the introduction of new technologies, can alter the relationship between algorithm inputs and observed outcomes. It is crucial to monitor these changes in input-output relationships because they may drive performance drift over time. Fourth, we limited our analysis to CAN version 2.5, the operational version for most of our study period, but acknowledge that newer versions (ie, CAN 3.0) may yield different results, while older versions could exaggerate drift, making our findings conservative with respect to model degradation. Lastly, we used only 1 CAN score per patient per year, calculated in December, due to data availability and computational constraints. Although this approach allows for consistent longitudinal comparisons, it does not account for intra-annual drift and seasonal fluctuations of the prevalence of the combined outcome.

## Conclusions

This cohort study found that nationally deployed clinical risk algorithms can improve health care delivery but are susceptible to gradual and sudden declines in operationally and clinically relevant performance metrics. Deterioration of algorithm performance may develop from changes in patient data and outcome rates. Performance drift undermines the reliability of quality metrics generated from algorithm classifications. Postmarket monitoring frameworks that track changes in algorithm performance as well as the mechanisms and impact of this drift should be prioritized.

## Notes

Supplement 1. **eTable 1.** Original algorithm covariates, corresponding variable description and type, odds ratios, and 95% confidence bounds on the odds ratios **eTable 2.** Use cases of clinical risk scores and hypothetical impact of performance drift **eTable 3.** VHA Stop Codes and CPT Codes corresponding to palliative care referrals **eFigure 1.** Performance drift in VA CAN across time period, by risk percentile threshold (50th, 70th, 95th, 98th, 99th) and classification metric **eTable 4.** Performance metric value, separated by year, metric, and threshold, with 95% confidence intervals **eTable 5.** Performance drift, measured by absolute change in metric (%), separated by time period, metric, and threshold, with 95% confidence intervals **eFigure 2.** Changes in the number of false positives identified due to drift, grouped by time period and threshold **eFigure 3.** Changes in the number of true positives identified due to drift, grouped by time period and threshold **eTable 6.** Classification rates, by year, metric, and threshold (50th, 70th, 95th, 98th, 99th), with 95% confidence intervals **eTable 7.** Possible covariate drivers of drift, with corresponding variable description, standardized mean difference (SMD) and variable ID for each drift time period **eFigure 4.** Covariate Shift in VA CAN covariates between 2019-2020, grouped by covariate category **eFigure 5.** Covariate Shift in VA CAN covariates between 2016-2018, grouped by covariate category **eTable 8.** Algorithm retraining, recalculated absolute drift (%) in 2016-2020 [2017-2021]* time period, 90th risk percentile threshold **eTable 9.** Clinical Impact Analysis, 98th and 99th percentile risk thresholds **eTable 10.** Model calibration and AUC between 2016[2017] and 2020[2021] **eAppendix 1.** Algorithm Classification and Performance Metrics **eAppendix 2.** Risk Distribution and Dataset Shift Mechanisms **eAppendix 3.** Standardized Mean Difference

Supplement 2. Data Sharing Statement
