# Supplementary material for: Performance Drift in a Nationally Deployed Population Health Risk Algorithm in the US Veterans Health Administration
Source: JAMA Health Forum. 2025 Aug 15;6(8):e252717. doi: 10.1001/jamahealthforum.2025.2717 (PMC12357188; doi:10.1001/jamahealthforum.2025.2717)
Supplement: Supplement 1. — eTable 1. Original algorithm covariates, corresponding variable description and type, odds ratios, and 95% confidence bounds on the odds ratios eTable 2. Use cases of clinical risk scores and hypothetical impact of performance drift eTable 3. VHA Stop Codes and CPT Codes corresponding to palliative care referrals eFigure 1. Performance drift in VA CAN across time period, by risk percentile threshold (50th, 70th, 95th, 98th, 99th) and classification metric eTable 4. Performance metric value, separated by year, metric, and threshold, with 95% confidence intervals eTable 5. Performance drift, measured by absolute change in metric (%), separated by time period, metric, and threshold, with 95% confidence intervals eFigure 2. Changes in the number of false positives identified due to drift, grouped by time period and threshold eFigure 3. Changes in the number of true positives identified due to drift, grouped by time period and threshold eTable 6. Classification rates, by year, metric, and threshold (50th, 70th, 95th, 98th, 99th), with 95% confidence intervals eTable 7. Possible covariate drivers of drift, with corresponding variable description, standardized mean difference (SMD) and variable ID for each drift time period eFigure 4. Covariate Shift in VA CAN covariates between 2019-2020, grouped by covariate category eFigure 5. Covariate Shift in VA CAN covariates between 2016-2018, grouped by covariate category eTable 8. Algorithm retraining, recalculated absolute drift (%) in 2016-2020 [2017-2021]* time period, 90th risk percentile threshold eTable 9. Clinical Impact Analysis, 98th and 99th percentile risk thresholds eTable 10. Model calibration and AUC between 2016[2017] and 2020[2021] eAppendix 1. Algorithm Classification and Performance Metrics eAppendix 2. Risk Distribution and Dataset Shift Mechanisms eAppendix 3. Standardized Mean Difference [file jamahealthforum-e252717-s001.pdf]

Supplemental Online Content

Kolla L, Linn K, Navathe AS, et al. Performance Drift in a Nationally Deployed Population Health Risk Algorithm in the US Veterans Health Administration. *JAMA Health Forum.* Published online August 15, 2025. doi:10.1001/jamahealthforum.2025.2717

eTable 1. Original algorithm covariates, corresponding variable description and type, odds ratios, and 95% confidence bounds on the odds ratios
eTable 2. Use cases of clinical risk scores and hypothetical impact of performance drift
eTable 3. VHA Stop Codes and CPT Codes corresponding to palliative care referrals
eFigure 1. Performance drift in VA CAN across time period, by risk percentile threshold (50th, 70th, 95th, 98th, 99th) and classification metric
eTable 4. Performance metric value, separated by year, metric, and threshold, with 95% confidence intervals
eTable 5. Performance drift, measured by absolute change in metric (%), separated by time period, metric, and threshold, with 95% confidence intervals
eFigure 2. Changes in the number of false positives identified due to drift, grouped by time period and threshold
eFigure 3. Changes in the number of true positives identified due to drift, grouped by time period and threshold
eTable 6. Classification rates, by year, metric, and threshold (50th, 70th, 95th, 98th, 99th), with 95% confidence intervals
eTable 7. Possible covariate drivers of drift, with corresponding variable description, standardized mean difference (SMD) and variable ID for each drift time period
eFigure 4. Covariate Shift in VA CAN covariates between 2019-2020, grouped by covariate category
eFigure 5. Covariate Shift in VA CAN covariates between 2016-2018, grouped by covariate category
eTable 8. Algorithm retraining, recalculated absolute drift (%) in 2016-2020 [2017-2021]* time period, 90th risk percentile threshold
eTable 9. Clinical Impact Analysis, 98th and 99th percentile risk thresholds
eTable 10. Model calibration and AUC between 2016[2017] and 2020[2021]
eAppendix 1. Algorithm Classification and Performance Metrics
eAppendix 2. Risk Distribution and Dataset Shift Mechanisms
eAppendix 3. Standardized Mean Difference

This supplemental material has been provided by the authors to give readers additional information about their work.

© 2025 Kolla L et al. *JAMA Health Forum*.

eTable 1: Original algorithm covariates, corresponding variable description and type, odds ratios, and 95% confidence bounds on the odds ratios

ID	Variable	Description	Type	Odds Ratio	OR_LB*	OR_UB*
1	agegrp_c1	Age < 55 vs Age >= 85	Demographic	0.373	0.348	0.401
2	agegrp_c2	Age 55-64 vs Age >= 85	Demographic	0.498	0.466	0.533
3	agegrp_c3	Age 65-74 vs Age >= 85	Demographic	0.494	0.462	0.527
4	agegrp_c4	Age 75-84 vs Age >= 85	Demographic	0.607	0.566	0.651
5	Albuminc0	Most Recent Albumin Lab result prior 1 year: > 3.4 vs No Albumin Lab	Laboratory	0.884	0.855	0.914
6	Albuminc1	Most Recent Albumin Lab result prior 1 year: 0-3.4 vs No Albumin Lab	Laboratory	1.263	1.217	1.311
7	AntiPsychotic0	No Antipsychotic Medication Filled prior 1 year vs Antipsychotic Med Filled	Pharmacy	0.834	0.815	0.852
8	BBC0	No BetaBlocker Medication Filled prior 1 year vs BetaBlocker Med Filled	Pharmacy	0.863	0.850	0.876
9	BUNC0	Most Recent Blood, Urine, Nitrogen Lab result prior 1 year: 0-16 vs No Lab	Laboratory	0.756	0.719	0.795
10	BUNC1	Most Recent Blood, Urine, Nitrogen Lab result prior 1 year: 17-25 vs No Lab	Laboratory	0.740	0.704	0.779
11	BUNC2	Most Recent Blood, Urine, Nitrogen Lab result prior 1 year: > 25 vs No Lab	Laboratory	0.909	0.861	0.960
12	CovWtFlag0	Coefficient of Variation for Weight: <= 4 vs > 4	Laboratory	0.764	0.754	0.775
13	CPT21to30Fon730_c0	Phone CPTs 21to30 mins prior 2 years: 0 vs > 1	Utilization	0.795	0.771	0.820
14	CPT21to30Fon730_c1	Phone CPTs 21to30 mins prior 2 years: 1 vs > 1	Utilization	0.923	0.893	0.954
15	cptCHSRay90_c0	Chest Xray CPTs prior 90 days: 0 vs > 2	Utilization	0.582	0.549	0.616
16	cptCHSRay90_c1	Chest Xray CPTs prior 90 days: 1-2 vs > 2	Utilization	0.900	0.849	0.954

© 2025 Kolla L et al. *JAMA Health Forum*.

17	CPToffVisEst90_c0	Established Office Visit CPTs prior 90 days: 0 vs > 4	Utilization	0.756	0.733	0.780
18	CPToffVisEst90_c1	Established Office Visit CPTs prior 90 days: 1-4 vs > 4	Utilization	0.797	0.774	0.820
19	Deyoc0	Charlson-Deyo Mortality Score: 0 vs > 4	Diagnosis	0.644	0.601	0.689
20	Deyoc1	Charlson-Deyo Mortality Score: 1 vs > 4	Diagnosis	0.771	0.720	0.825
21	Deyoc2	Charlson-Deyo Mortality Score: 2 vs > 4	Diagnosis	0.859	0.802	0.920
22	Deyoc3	Charlson-Deyo Mortality Score: 3-4 vs > 4	Diagnosis	0.900	0.841	0.962
23	diastc1	Most Recent Diastolic Blood Pressure Measurement prior 1 year: < 60 vs >= 90	Laboratory	0.993	0.947	1.043
24	diastc2	Most Recent Diastolic Blood Pressure Measurement prior 1 year: 60 < diast < 90 or Unknown vs >= 90	Laboratory	0.895	0.859	0.932
25	DxAlcohol730_c0	Alcohol Dxs prior 2 years: 0-1 vs > 1	Diagnosis	0.717	0.703	0.731
26	DxChrAirObstnec730_c0	Chronic Airway Obstruction Dxs prior 2 years: 0 vs > 0	Diagnosis	0.840	0.827	0.854
27	er1yc0	Emergency Room Stop Code Visits prior 1 year: 0 vs > 2	Utilization	0.682	0.660	0.704
28	er1yc1	Emergency Room Stop Code Visits prior 1 year: 1-2 vs > 2	Utilization	0.869	0.842	0.896
29	Furosemide0	No Furosemide Medication Filled prior 1 year vs Furosemide Med Filled	Pharmacy	0.885	0.868	0.903
30	HCC_Dementia0	No Dementia Dx vs Dementia Dx prior 1 year	Diagnosis	0.733	0.717	0.750
31	HCC_HxCHF0	No Congestive Heart Failure Dx vs Congestive Heart Failure Dx prior 1 year	Diagnosis	0.801	0.785	0.818

© 2025 Kolla L et al. *JAMA Health Forum*.

32	HCC_MetasCa0	No Metastatic Cancer Dx vs Metastatic Caner Dx prior 1 year	Diagnosis	0.780	0.763	0.797
33	HmgCoa0	No Hydroxymethylglutarly-CoenzymeA Medication Filled prior 1 year vs HMGCOA Med Filled	Pharmacy	1.130	1.114	1.146
34	hospbedC0	Hosp Stays & Bed Days of Care prior 1 year: level 0 vs 4	Utilization	1.915	1.614	2.271
35	hospbedC1	Hosp Stays & Bed Days of Care prior 1 year: level 1 vs 4	Utilization	1.284	1.087	1.516
36	hospbedC2	Hosp Stays & Bed Days of Care prior 1 year: level 2 vs 4	Utilization	1.249	1.039	1.501
37	hospbedC3	Hosp Stays & Bed Days of Care prior 1 year: level 3 vs 4	Utilization	1.028	0.867	1.219
38	i_admtAll365_c0	All Hospital Admissions prior 1 year: 0 vs > 2	Utilization	0.301	0.270	0.336
39	i_admtAll365_c1	All Hospital Admissions prior 1 year: 1 vs > 2	Utilization	0.530	0.476	0.590
40	i_admtAll365_c2	All Hospital Admissions prior 1 year: 2 vs > 2	Utilization	0.708	0.636	0.787
41	LbLymphosbpl365_c0	Low Lymphocites Labs prior 1 year: 0 vs > 1	Laboratory	0.932	0.900	0.965
42	LbLymphosbpl365_c1	Low Lymphocites Labs prior 1 year: 1 vs > 1	Laboratory	1.061	1.022	1.102
43	LbRBCsynfll730_c0	Low Red Blood Count Labs prior 2 years: 0 vs > 9	Laboratory	0.833	0.806	0.861
44	LbRBCsynfll730_c1	Low Red Blood Count Labs prior 2 years: 1-9 vs > 9	Laboratory	1.029	0.997	1.062
45	lbWBCspbH730_C0	High White Blood Cell Labs prior 2 years: 0 vs > 1	Laboratory	0.849	0.821	0.878
46	lbWBCspbH730_C1	High White Blood Cell Labs prior 2 years: 1 vs > 1	Laboratory	0.975	0.939	1.012
47	Married0	Not Married vs Married	Demographic	1.172	1.157	1.188
48	MentPTSD0	No Mental Disorder Dx & No PTSD Dx vs Mental	Diagnosis	0.890	0.860	0.920

© 2025 Kolla L et al. *JAMA Health Forum*.

		Disorder Dx or PTSD Dx prior 1 year				
49	MentPTSD1	Mental Disorder Dx & PTSD Dx vs Mental Disorder Dx or PTSD Dx prior 1 year	Diagnosis	0.930	0.897	0.963
50	nface1yC0	Other Non-Face Stop Code Visits prior 1 year: 0 vs > 15	Utilization	0.671	0.588	0.766
51	nface1yC1	Other Non-Face Stop Code Visits prior 1 year: 1-2 vs > 15	Utilization	0.659	0.583	0.744
52	nface1yC2	Other Non-Face Stop Code Visits prior 1 year: 3-4 vs > 15	Utilization	0.719	0.637	0.812
53	nface1yC3	Other Non-Face Stop Code Visits prior 1 year: 5-9 vs > 15	Utilization	0.850	0.757	0.956
54	nface1yC4	Other Non-Face Stop Code Visits prior 1 year: 10-15 vs > 15	Utilization	0.937	0.832	1.055
55	Prior1245_0	Vet Priority level 0 vs 5	Demographic	0.864	0.793	0.943
56	Prior1245_1	Vet Priority level 1 vs 5	Demographic	0.966	0.885	1.054
57	Prior1245_2	Vet Priority level 2 vs 5	Demographic	0.808	0.739	0.883
58	Prior1245_4	Vet Priority level 4 vs 5	Demographic	1.005	0.906	1.115
59	Pulsec1	Most Recent Pulse Vital Measurement prior 1 year: < 60 vs >= 90	Laboratory	0.689	0.663	0.716
60	Pulsec2	Most Recent Pulse Vital Measurement prior 1 year: 60 <= pulse < 90 or Unknown vs >= 90	Laboratory	0.795	0.769	0.822
61	Respc1	Most Recent Respiration Vital Measurement prior 1 year: < 18 vs >= 20	Laboratory	0.923	0.898	0.948
62	Respc2	Most Recent Respiration Vital Measurement prior 1 year: 18 <= respir < 20 or Unknown vs >= 20	Laboratory	0.957	0.933	0.982
63	sc_CT365_c0	CT Primary Stop Code Visits prior 1 year: 0 vs > 3	Utilization	0.823	0.795	0.851
64	sc_CT365_c1	CT Primary Stop Code Visits prior 1 year: 1-3 vs > 3	Utilization	0.945	0.914	0.978

© 2025 Kolla L et al. *JAMA Health Forum*.

65	ses_index_decile_1	Default/No SES Index Calculated vs SES Index Decile 10	Demographic	1.121	0.908	1.384
66	ses_index_decile0	SES Index Decile 1 vs SES Index Decile 10	Demographic	1.175	0.951	1.452
67	ses_index_decile1	SES Index Decile 2 vs SES Index Decile 10	Demographic	1.136	0.919	1.405
68	ses_index_decile2	SES Index Decile 3 vs SES Index Decile 10	Demographic	1.117	0.903	1.383
69	ses_index_decile3	SES Index Decile 4 vs SES Index Decile 10	Demographic	1.106	0.893	1.370
70	ses_index_decile4	SES Index Decile 5 vs SES Index Decile 10	Demographic	1.067	0.861	1.322
71	ses_index_decile5	SES Index Decile 6 vs SES Index Decile 10	Demographic	1.063	0.858	1.317
72	ses_index_decile6	SES Index Decile 7 vs SES Index Decile 10	Demographic	1.058	0.853	1.311
73	ses_index_decile7	SES Index Decile 8 vs SES Index Decile 10	Demographic	1.050	0.847	1.302
74	ses_index_decile8	SES Index Decile 9 vs SES Index Decile 10	Demographic	1.019	0.822	1.265
75	Systc1	Most Recent Systolic Blood Pressure Measurement prior 1 year: < 110 vs >= 160	Laboratory	1.077	1.016	1.142
76	Systc2	Most Recent Systolic Blood Pressure Measurement prior 1 year: 110 <= syst < 140 or Unknown vs >= 160	Laboratory	0.884	0.840	0.932
77	Systc3	Most Recent Systolic Blood Pressure Measurement prior 1 year: 140 <= syst < 160 vs >= 160	Laboratory	0.919	0.871	0.971
**78**	**visits_c0**	Outpatient Visits prior 3 years: 0-90 vs > 180	Utilization	0.596	0.575	0.618
**79**	**visits_c1**	Outpatient Visits prior 3 years: 91-180 vs > 180	Utilization	0.839	0.811	0.867

*OR = Odds Ratio ; LB = Lower Bound (95% CI); UB = Upper Bound (95% CI)

© 2025 Kolla L et al. *JAMA Health Forum*.

eTable 2 -Use cases of clinical risk scores and hypothetical impact of performance drift

Use Case	Description	Reference	Impact of Drift
Quality Metric (Benchmarking)	Providers and hospitals are held accountable to care quality benchmarks (e.g., readmission rates, mortality), and risk prediction scores help ensure these benchmarks are appropriate for the patient population. Providers can use these scores to assess whether their performance aligns with expected outcomes based on patient risk profiles.	[1,2]	Can lead to inaccurate benchmarks and an inability for providers and hospitals to evaluate their care quality effectively.
Risk Adjustment	Algorithm-generated risk prediction scores adjust quality metrics for varying patient risk profiles, ensuring fair comparisons of outcomes (e.g., mortality, readmission) across hospitals or providers.	[3,4]	Can lead to skewing of risk-adjusted quality metrics and unfair comparison of hospitals or providers with differing patient populations, thereby undermining the validity of outcome assessments.
Payment Algorithms and Insurance	In value-based care, risk prediction scores adjust payments to reflect patient risk. For example, providers may get higher reimbursement for managing higher-risk patients, which aligns financial incentives with outcomes. Insurers can use these scores to adjust premiums or coverage (e.g., CMS's Hospital Readmission Reduction Program (HRRP)).	[5]	Could lead to inaccurate adjustments in value-based care reimbursements, unfair insurance premiums, and penalties in programs like HRRP, ultimately distorting financial and quality assessments.
Resource Allocation	By predicting patient outcomes, risk scores can inform the allocation of limited healthcare resources, ensuring that interventions are directed toward patients most likely to benefit. This would lead to more efficient use of hospital beds, staff time, and medical treatments, while reducing unnecessary tests, treatments, or	[6]	Can result in inefficient allocation of healthcare resources, overuse of interventions for low-risk patients, and missed opportunities to focus care for high-risk patients.

© 2025 Kolla L et al. *JAMA Health Forum*.

	hospitalizations for low-risk patients.		
Algorithm Driven Alerts and Decision Support	Hospitals integrate risk scores into EHR systems to trigger clinical alerts, such as sepsis risk, prompting timely tests or treatments. These scores also enhance clinical decision support tools by providing automated recommendations based on patient risk and conditions.	[7,8]	Can lead to missed opportunities for timely preventive interventions or chronic disease management, thereby increasing the risk of adverse health outcomes and avoidable healthcare utilization.
Preventative Care and Population Management	In outpatient care, risk scores can prioritize patients for additional preventative interventions, such as lifestyle counseling for high cardiovascular risk, and help manage chronic diseases by prompting timely interventions to prevent complications or hospitalizations.	[9]	Can lead to missed preventive interventions or delayed management of chronic diseases, increasing the likelihood of complications and avoidable healthcare costs.
Informed clinical decision-making	Clinicians use risk scores to guide targeted interventions, such as early antibiotics for high sepsis risk or aggressive treatment for high cardiovascular risk. High mortality risk scores can prompt serious illness conversations on end-of-life care or palliative care.	[10–12]	Can result in delayed or inappropriate targeted interventions and missed opportunities for timely serious illness conversations.
Risk Stratification and Triage	Risk scores help healthcare providers classify patients based on the likelihood of adverse outcomes (e.g., mortality, hospitalization). This stratification allows for prioritization in high-demand settings, such as emergency departments (for triage).	[13]	Can lead to misprioritization of patients in high-demand settings and suboptimal decisions regarding inpatient management, level of care, and discharge planning.

Work Cited:

1. Subbe C, Jishi F, Hibbs R. The Simple Clinical Score: a tool for benchmarking of emergency admissions in acute internal medicine. *Clin Med*. 2010;10(4):352-357. doi:10.7861/clinmedicine.10-4-352

© 2025 Kolla L et al. *JAMA Health Forum*.

2. Darby JL, Davis BS, Barbash IJ, Kahn JM. An administrative model for benchmarking hospitals on their 30-day sepsis mortality. *BMC Health Serv Res*. 2019;19(1):221. doi:10.1186/s12913-019-4037-x

3. Murtaugh CM. Risk Adjustment and Public Reporting on Home Health Care. *Health Care Financ Rev*. 28(3).

4. Zink A, Rose S. Identifying undercompensated groups defined by multiple attributes in risk adjustment. *BMJ Health Care Inform*. 2021;28(1):e100414. doi:10.1136/bmjhci-2021-100414

5. Hospital Readmissions Reduction Program (HRRP) | NEJM Catalyst.

6. Gail MH, Pee D. Robustness of risk-based allocation of resources for disease prevention. *Stat Methods Med Res*. 2020;29(12):3511-3524. doi:10.1177/0962280220930055

7. Wong A, Cao J, Lyons PG, et al. Quantification of Sepsis Model Alerts in 24 US Hospitals Before and During the COVID-19 Pandemic. *JAMA Netw Open*. 2021;4(11):e2135286. doi:10.1001/jamanetworkopen.2021.35286

8. Wong A, Otles E, Donnelly JP, et al. External Validation of a Widely Implemented Proprietary Sepsis Prediction Model in Hospitalized Patients. *JAMA Intern Med*. 2021;181(8):1065. doi:10.1001/jamainternmed.2021.2626

9. Oddy C, Zhang J, Morley J, Ashrafian H. Promising algorithms to perilous applications: a systematic review of risk stratification tools for predicting healthcare utilisation. *BMJ Health Care Inform*. 2024;31(1):e101065. doi:10.1136/bmjhci-2024-101065

10. Parikh RB, Zhang Y, Kolla L, et al. Performance drift in a mortality prediction algorithm among patients with cancer during the SARS-CoV-2 pandemic. *J Am Med Inform Assoc*. 2023;30(2):348-354. doi:10.1093/jamia/ocac221

11. Parikh RB, Manz C, Chivers C, et al. Machine Learning Approaches to Predict 6-Month Mortality Among Patients With Cancer. *JAMA Netw Open*. 2019;2(10):e1915997. doi:10.1001/jamanetworkopen.2019.15997

12. Manz CR, Parikh RB, Small DS, et al. Effect of Integrating Machine Learning Mortality Estimates With Behavioral Nudges to Clinicians on Serious Illness Conversations Among Patients With Cancer: A Stepped-Wedge Cluster Randomized Clinical Trial. *JAMA Oncol*. 2020;6(12):e204759. doi:10.1001/jamaoncol.2020.4759

13. Tyler S, Olis M, Aust N, et al. Use of Artificial Intelligence in Triage in Hospital Emergency Departments: A Scoping Review. *Cureus*. Published online May 8, 2024. doi:10.7759/cureus.59906

© 2025 Kolla L et al. *JAMA Health Forum*.

eTable 3 – VHA Stop Codes and CPT Codes corresponding to palliative care referrals

The number of available palliative care consultations or referrals were calculated by extracting and merging the following CPT codes and DSS identifiers: CPT codes (99241-99245, 99251-99255, 99201-99205, or 99221-99223) and DSS identifiers (351 or 353. Extracted palliative care referrals were binned by fiscal year, where each fiscal year contains referrals between October 1st of a specified year to September 30th of the following year. Duplicated referrals were removed and only one referral per fiscal year was chosen. Importantly, receiving a palliative care referral does not affect the combined outcome for a given patient as this algorithm did not directly influence patient care decisions.

StopCode	StopCodeName
351	HOSPICE CARE
353	PALLIATIVE CARE

CPTCode	CPTName	CPTDescription	CPTCategory
99201	OFFICE/OUTPATIENT VISIT NEW	OFFICE OR OTHER OUTPATIENT VISIT FOR THE EVALUATION AND MANAGEMENT OF A NEW PATIENT, WHICH REQUIRES THESE 3 KEY COMPONENTS: A PROBLEM FOCUSED HISTORY; A PROBLEM FOCUSED EXAMINATION; STRAIGHTFORWARD MEDICAL DECISION MAKING. COUNSELING AND/OR COORDINATION OF CARE WITH OTHER PHYSICIANS, OTHER QUALIFIED HEALTH CARE PROFESSIONALS, OR AGENCIES ARE PROVIDED CONSISTENT WITH THE NATURE OF THE PROBLEM(S) AND THE PATIENT'S AND/OR FAMILY'S NEEDS. USUALLY, THE PRESENTING PROBLEM(S) ARE SELF LIMITED OR MINOR. TYPICALLY, 10 MINUTES ARE SPENT FACE-TO-FACE WITH THE PATIENT AND/OR FAMILY.	OFFICE OR OTHER OUTPATIENT SERVICES
99202	OFFICE O/P NEW SF 15-29 MIN	OFFICE OR OTHER OUTPATIENT VISIT FOR THE EVALUATION AND MANAGEMENT OF A NEW PATIENT, WHICH REQUIRES A MEDICALLY APPROPRIATE HISTORY AND/OR EXAMINATION AND STRAIGHTFORWARD MEDICAL DECISION MAKING. WHEN USING TIME FOR CODE SELECTION, 15-29 MINUTES OF TOTAL TIME IS SPENT ON THE DATE OF THE ENCOUNTER.	OFFICE OR OTHER OUTPATIENT SERVICES
99203	OFFICE O/P NEW LOW 30-44 MIN	OFFICE OR OTHER OUTPATIENT VISIT FOR THE EVALUATION AND MANAGEMENT OF A NEW PATIENT, WHICH REQUIRES A MEDICALLY APPROPRIATE HISTORY AND/OR EXAMINATION AND LOW LEVEL OF MEDICAL DECISION MAKING. WHEN USING TIME FOR CODE SELECTION, 30-44 MINUTES OF TOTAL TIME IS SPENT ON THE DATE OF THE ENCOUNTER.	OFFICE OR OTHER OUTPATIENT SERVICES

© 2025 Kolla L et al. *JAMA Health Forum*.

99204	OFFICE O/P NEW MOD 45-59 MIN	OFFICE OR OTHER OUTPATIENT VISIT FOR THE EVALUATION AND MANAGEMENT OF A NEW PATIENT, WHICH REQUIRES A MEDICALLY APPROPRIATE HISTORY AND/OR EXAMINATION AND MODERATE LEVEL OF MEDICAL DECISION MAKING. WHEN USING TIME FOR CODE SELECTION, 45-59 MINUTES OF TOTAL TIME IS SPENT ON THE DATE OF THE ENCOUNTER.	OFFICE OR OTHER OUTPATIENT SERVICES
99205	OFFICE O/P NEW HI 60-74 MIN	OFFICE OR OTHER OUTPATIENT VISIT FOR THE EVALUATION AND MANAGEMENT OF A NEW PATIENT, WHICH REQUIRES A MEDICALLY APPROPRIATE HISTORY AND/OR EXAMINATION AND HIGH LEVEL OF MEDICAL DECISION MAKING. WHEN USING TIME FOR CODE SELECTION, 60-74 MINUTES OF TOTAL TIME IS SPENT ON THE DATE OF THE ENCOUNTER.	OFFICE OR OTHER OUTPATIENT SERVICES
99221	INITIAL HOSPITA L CARE	INITIAL HOSPITAL CARE, PER DAY, FOR THE EVALUATION AND MANAGEMENT OF A PATIENT, WHICH REQUIRES THESE 3 KEY COMPONENTS: A DETAILED OR COMPREHENSIVE HISTORY; A DETAILED OR COMPREHENSIVE EXAMINATION; AND MEDICAL DECISION MAKING THAT IS STRAIGHTFORWARD OR OF LOW COMPLEXITY. COUNSELING AND/OR COORDINATION OF CARE WITH OTHER PHYSICIANS, OTHER QUALIFIED HEALTH CARE PROFESSIONALS, OR AGENCIES ARE PROVIDED CONSISTENT WITH THE NATURE OF THE PROBLEM(S) AND THE PATIENT'S AND/OR FAMILY'S NEEDS. USUALLY, THE PROBLEM(S) REQUIRING ADMISSION ARE OF LOW SEVERITY. TYPICALLY, 30 MINUTES ARE SPENT AT THE BEDSIDE AND ON THE PATIENT'S HOSPITAL FLOOR OR UNIT.	HOSPITAL INPATIENT SERVICES
99222	INITIAL HOSPITA L CARE	INITIAL HOSPITAL CARE, PER DAY, FOR THE EVALUATION AND MANAGEMENT OF A PATIENT, WHICH REQUIRES THESE 3 KEY COMPONENTS: A COMPREHENSIVE HISTORY; A COMPREHENSIVE EXAMINATION; AND MEDICAL DECISION MAKING OF MODERATE COMPLEXITY. COUNSELING AND/OR COORDINATION OF CARE WITH OTHER PHYSICIANS, OTHER QUALIFIED HEALTH CARE PROFESSIONALS, OR AGENCIES ARE PROVIDED CONSISTENT WITH THE NATURE OF THE PROBLEM(S) AND THE PATIENT'S AND/OR FAMILY'S NEEDS. USUALLY, THE PROBLEM(S) REQUIRING ADMISSION ARE OF MODERATE SEVERITY. TYPICALLY, 50 MINUTES ARE SPENT AT THE BEDSIDE AND ON THE PATIENT'S HOSPITAL FLOOR OR UNIT.	HOSPITAL INPATIENT SERVICES
99223	INITIAL HOSPITA L CARE	INITIAL HOSPITAL CARE, PER DAY, FOR THE EVALUATION AND MANAGEMENT OF A PATIENT, WHICH REQUIRES THESE 3 KEY COMPONENTS: A COMPREHENSIVE HISTORY; A COMPREHENSIVE	HOSPITAL INPATIENT SERVICES

© 2025 Kolla L et al. *JAMA Health Forum*.

		EXAMINATION; AND MEDICAL DECISION MAKING OF HIGH COMPLEXITY. COUNSELING AND/OR COORDINATION OF CARE WITH OTHER PHYSICIANS, OTHER QUALIFIED HEALTH CARE PROFESSIONALS, OR AGENCIES ARE PROVIDED CONSISTENT WITH THE NATURE OF THE PROBLEM(S) AND THE PATIENT'S AND/OR FAMILY'S NEEDS. USUALLY, THE PROBLEM(S) REQUIRING ADMISSION ARE OF HIGH SEVERITY. TYPICALLY, 70 MINUTES ARE SPENT AT THE BEDSIDE AND ON THE PATIENT'S HOSPITAL FLOOR OR UNIT.	
99241	OFFICE CONSULTATION	OFFICE CONSULTATION FOR A NEW OR ESTABLISHED PATIENT, WHICH REQUIRES THESE 3 KEY COMPONENTS: A PROBLEM FOCUSED HISTORY; A PROBLEM FOCUSED EXAMINATION; AND STRAIGHTFORWARD MEDICAL DECISION MAKING. COUNSELING AND/OR COORDINATION OF CARE WITH OTHER PHYSICIANS, OTHER QUALIFIED HEALTH CARE PROFESSIONALS, OR AGENCIES ARE PROVIDED CONSISTENT WITH THE NATURE OF THE PROBLEM(S) AND THE PATIENT'S AND/OR FAMILY'S NEEDS. USUALLY, THE PRESENTING PROBLEM(S) ARE SELF LIMITED OR MINOR. TYPICALLY, 15 MINUTES ARE SPENT FACE-TO-FACE WITH THE PATIENT AND/OR FAMILY.	CONSULTATIONS
99242	OFFICE CONSULTATION	OFFICE CONSULTATION FOR A NEW OR ESTABLISHED PATIENT, WHICH REQUIRES THESE 3 KEY COMPONENTS: AN EXPANDED PROBLEM FOCUSED HISTORY; AN EXPANDED PROBLEM FOCUSED EXAMINATION; AND STRAIGHTFORWARD MEDICAL DECISION MAKING. COUNSELING AND/OR COORDINATION OF CARE WITH OTHER PHYSICIANS, OTHER QUALIFIED HEALTH CARE PROFESSIONALS, OR AGENCIES ARE PROVIDED CONSISTENT WITH THE NATURE OF THE PROBLEM(S) AND THE PATIENT'S AND/OR FAMILY'S NEEDS. USUALLY, THE PRESENTING PROBLEM(S) ARE OF LOW SEVERITY. TYPICALLY, 30 MINUTES ARE SPENT FACE-TO-FACE WITH THE PATIENT AND/OR FAMILY.	CONSULTATIONS
99243	OFFICE CONSULTATION	OFFICE CONSULTATION FOR A NEW OR ESTABLISHED PATIENT, WHICH REQUIRES THESE 3 KEY COMPONENTS: A DETAILED HISTORY; A DETAILED EXAMINATION; AND MEDICAL DECISION MAKING OF LOW COMPLEXITY. COUNSELING AND/OR COORDINATION OF CARE WITH OTHER PHYSICIANS, OTHER QUALIFIED HEALTH CARE PROFESSIONALS, OR AGENCIES ARE PROVIDED CONSISTENT WITH THE NATURE OF THE PROBLEM(S) AND THE PATIENT'S AND/OR FAMILY'S NEEDS. USUALLY, THE PRESENTING PROBLEM(S) ARE OF MODERATE SEVERITY. TYPICALLY, 40	CONSULTATIONS

© 2025 Kolla L et al. *JAMA Health Forum*.

		MINUTES ARE SPENT FACE-TO-FACE WITH THE PATIENT AND/OR FAMILY.	
99244	OFFICE CONSULTATION	OFFICE CONSULTATION FOR A NEW OR ESTABLISHED PATIENT, WHICH REQUIRES THESE 3 KEY COMPONENTS: A COMPREHENSIVE HISTORY; A COMPREHENSIVE EXAMINATION; AND MEDICAL DECISION MAKING OF MODERATE COMPLEXITY. COUNSELING AND/OR COORDINATION OF CARE WITH OTHER PHYSICIANS, OTHER QUALIFIED HEALTH CARE PROFESSIONALS, OR AGENCIES ARE PROVIDED CONSISTENT WITH THE NATURE OF THE PROBLEM(S) AND THE PATIENT'S AND/OR FAMILY'S NEEDS. USUALLY, THE PRESENTING PROBLEM(S) ARE OF MODERATE TO HIGH SEVERITY. TYPICALLY, 60 MINUTES ARE SPENT FACE-TO-FACE WITH THE PATIENT AND/OR FAMILY.	CONSULTATIONS
99245	OFFICE CONSULTATION	OFFICE CONSULTATION FOR A NEW OR ESTABLISHED PATIENT, WHICH REQUIRES THESE 3 KEY COMPONENTS: A COMPREHENSIVE HISTORY; A COMPREHENSIVE EXAMINATION; AND MEDICAL DECISION MAKING OF HIGH COMPLEXITY. COUNSELING AND/OR COORDINATION OF CARE WITH OTHER PHYSICIANS, OTHER QUALIFIED HEALTH CARE PROFESSIONALS, OR AGENCIES ARE PROVIDED CONSISTENT WITH THE NATURE OF THE PROBLEM(S) AND THE PATIENT'S AND/OR FAMILY'S NEEDS. USUALLY, THE PRESENTING PROBLEM(S) ARE OF MODERATE TO HIGH SEVERITY. TYPICALLY, 80 MINUTES ARE SPENT FACE-TO-FACE WITH THE PATIENT AND/OR FAMILY.	CONSULTATIONS
99251	INPATIENT CONSULTATION	INPATIENT CONSULTATION FOR A NEW OR ESTABLISHED PATIENT, WHICH REQUIRES THESE 3 KEY COMPONENTS: A PROBLEM FOCUSED HISTORY; A PROBLEM FOCUSED EXAMINATION; AND STRAIGHTFORWARD MEDICAL DECISION MAKING. COUNSELING AND/OR COORDINATION OF CARE WITH OTHER PHYSICIANS, OTHER QUALIFIED HEALTH CARE PROFESSIONALS, OR AGENCIES ARE PROVIDED CONSISTENT WITH THE NATURE OF THE PROBLEM(S) AND THE PATIENT'S AND/OR FAMILY'S NEEDS. USUALLY, THE PRESENTING PROBLEM(S) ARE SELF LIMITED OR MINOR. TYPICALLY, 20 MINUTES ARE SPENT AT THE BEDSIDE AND ON THE PATIENT'S HOSPITAL FLOOR OR UNIT.	CONSULTATIONS
99252	INPATIENT CONSULTATION	INPATIENT CONSULTATION FOR A NEW OR ESTABLISHED PATIENT, WHICH REQUIRES THESE 3 KEY COMPONENTS: AN EXPANDED PROBLEM FOCUSED HISTORY; AN EXPANDED PROBLEM FOCUSED EXAMINATION; AND STRAIGHTFORWARD MEDICAL DECISION MAKING. COUNSELING AND/OR COORDINATION OF CARE WITH OTHER PHYSICIANS,	CONSULTATIONS

© 2025 Kolla L et al. *JAMA Health Forum*.

		OTHER QUALIFIED HEALTH CARE PROFESSIONALS, OR AGENCIES ARE PROVIDED CONSISTENT WITH THE NATURE OF THE PROBLEM(S) AND THE PATIENT'S AND/OR FAMILY'S NEEDS. USUALLY, THE PRESENTING PROBLEM(S) ARE OF LOW SEVERITY. TYPICALLY, 40 MINUTES ARE SPENT AT THE BEDSIDE AND ON THE PATIENT'S HOSPITAL FLOOR OR UNIT.	
99253	INPATIEN T CONSULT ATION	INPATIENT CONSULTATION FOR A NEW OR ESTABLISHED PATIENT, WHICH REQUIRES THESE 3 KEY COMPONENTS: A DETAILED HISTORY; A DETAILED EXAMINATION; AND MEDICAL DECISION MAKING OF LOW COMPLEXITY. COUNSELING AND/OR COORDINATION OF CARE WITH OTHER PHYSICIANS, OTHER QUALIFIED HEALTH CARE PROFESSIONALS, OR AGENCIES ARE PROVIDED CONSISTENT WITH THE NATURE OF THE PROBLEM(S) AND THE PATIENT'S AND/OR FAMILY'S NEEDS. USUALLY, THE PRESENTING PROBLEM(S) ARE OF MODERATE SEVERITY. TYPICALLY, 55 MINUTES ARE SPENT AT THE BEDSIDE AND ON THE PATIENT'S HOSPITAL FLOOR OR UNIT.	CONSULTATIO NS
99254	INPATIEN T CONSULT ATION	INPATIENT CONSULTATION FOR A NEW OR ESTABLISHED PATIENT, WHICH REQUIRES THESE 3 KEY COMPONENTS: A COMPREHENSIVE HISTORY; A COMPREHENSIVE EXAMINATION; AND MEDICAL DECISION MAKING OF MODERATE COMPLEXITY. COUNSELING AND/OR COORDINATION OF CARE WITH OTHER PHYSICIANS, OTHER QUALIFIED HEALTH CARE PROFESSIONALS, OR AGENCIES ARE PROVIDED CONSISTENT WITH THE NATURE OF THE PROBLEM(S) AND THE PATIENT'S AND/OR FAMILY'S NEEDS. USUALLY, THE PRESENTING PROBLEM(S) ARE OF MODERATE TO HIGH SEVERITY. TYPICALLY, 80 MINUTES ARE SPENT AT THE BEDSIDE AND ON THE PATIENT'S HOSPITAL FLOOR OR UNIT.	CONSULTATIO NS
99255	INPATIEN T CONSULT ATION	INPATIENT CONSULTATION FOR A NEW OR ESTABLISHED PATIENT, WHICH REQUIRES THESE 3 KEY COMPONENTS: A COMPREHENSIVE HISTORY; A COMPREHENSIVE EXAMINATION; AND MEDICAL DECISION MAKING OF HIGH COMPLEXITY. COUNSELING AND/OR COORDINATION OF CARE WITH OTHER PHYSICIANS, OTHER QUALIFIED HEALTH CARE PROFESSIONALS, OR AGENCIES ARE PROVIDED CONSISTENT WITH THE NATURE OF THE PROBLEM(S) AND THE PATIENT'S AND/OR FAMILY'S NEEDS. USUALLY, THE PRESENTING PROBLEM(S) ARE OF MODERATE TO HIGH SEVERITY. TYPICALLY, 110 MINUTES ARE SPENT AT THE BEDSIDE AND ON THE PATIENT'S HOSPITAL FLOOR OR UNIT.	CONSULTATIO NS

© 2025 Kolla L et al. *JAMA Health Forum*.

eFigure 1: Performance drift in VA CAN across time period, by risk percentile threshold (50th, 70th, 95th, 98th, 99th) and classification metric

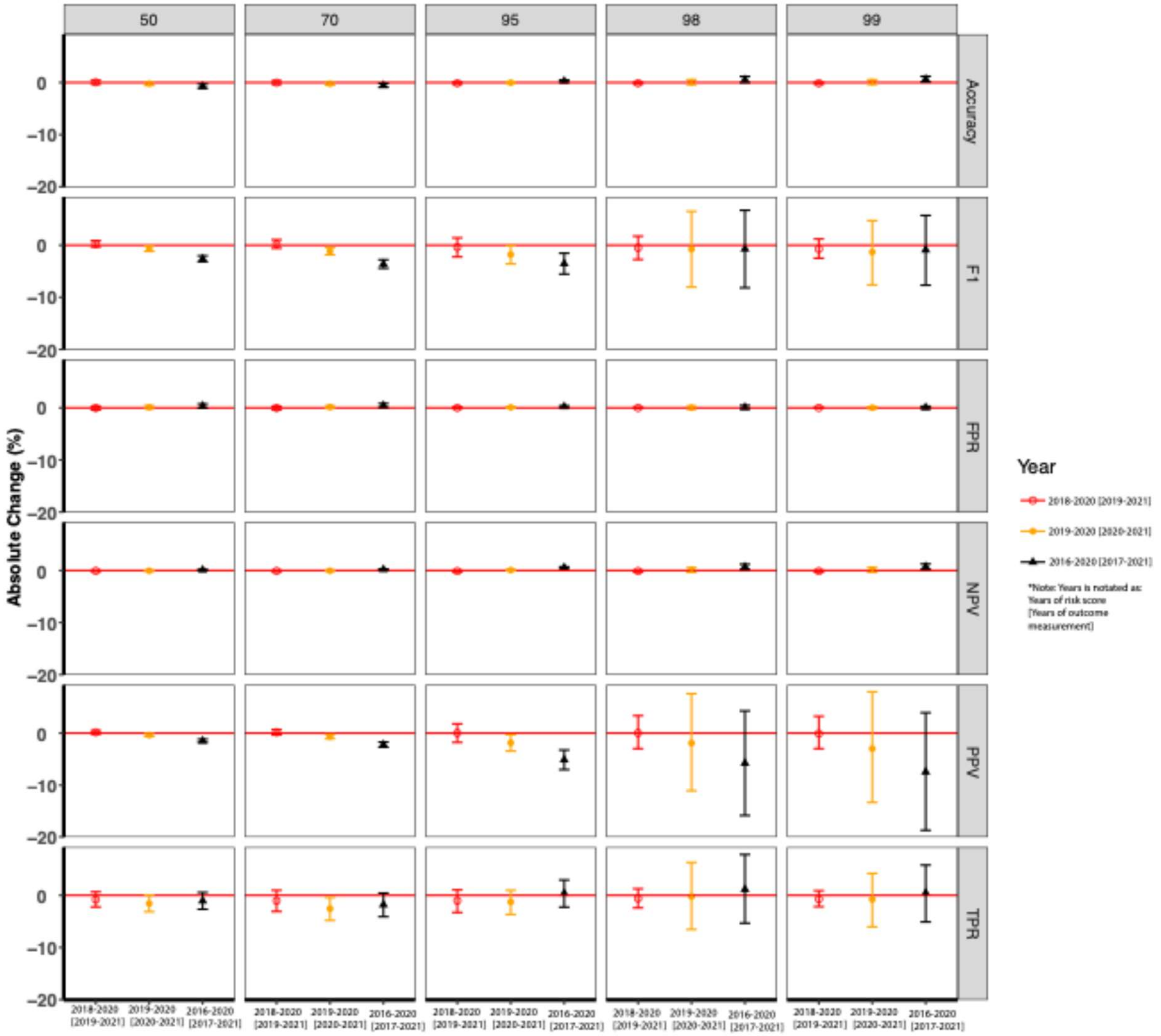

Caption: Figure presents performance drift across six metrics (i.e. AUC, F1, FPR, NPV, PPV, TPR). Performance drift was measured as the absolute percentage change in a given performance metric (y-axis) from baseline metrics and was calculated for three different time periods (x-axis): 2016–2018 [2017–2019], 2019–2020 [2020–2021], and 2016–2020 [2017–2021]. Baseline metrics were derived from 2016, 2019, and 2016 for the three time periods, respectively. Years are notated as "Years of risk score [Years of outcome measurement]". Results are stratified by percentile risk threshold (i.e. 50th, 70th, 95th, 98th , 99th) in the vertical columns of the figure. The solid red horizontal line at y=0 represents no change in the metric across the time period. 95% confidence intervals surround each performance drift point estimate. Trends in point estimates of drift are similar across thresholds. Higher thresholds have larger confidence intervals due to limited number of cases included in the high-risk group.

© 2025 Kolla L et al. *JAMA Health Forum*.

eTable 4: Performance metric value, separated by year, metric, and threshold, with 95% confidence intervals

Year*	Metric**	Threshold	Performance Metric (%)	Lower Bound	Upper Bound
2016 [2017]	Accuracy	50	53.0013685	52.6821839	53.3348241
2016 [2017]	Accuracy	70	72.0223483	71.6995702	72.3180963
2016 [2017]	Accuracy	90	89.8883929	89.6922538	90.0853379
2016 [2017]	Accuracy	95	93.7713158	93.6073654	93.9284855
2016 [2017]	Accuracy	98	95.6506578	95.5112642	95.7887309
2016 [2017]	Accuracy	99	96.1252934	95.9958592	96.2509598
2016 [2017]	F1	50	12.5869104	12.1760207	12.9912856
2016 [2017]	F1	70	17.1495487	16.5744419	17.7448681
2016 [2017]	F1	90	26.5600042	25.5244424	27.5892308
2016 [2017]	F1	95	28.9635958	27.5945286	30.2943918
2016 [2017]	F1	98	24.6032239	23.0442693	26.111307
2016 [2017]	F1	99	39.3931649	38.0235832	40.6730929
2016 [2017]	FPR	50	48.4392765	48.0997655	48.7610251
2016 [2017]	FPR	70	28.1661459	27.8713659	28.4884184
2016 [2017]	FPR	90	8.49150624	8.30264406	8.68289871
2016 [2017]	FPR	95	3.87599078	3.74145623	4.00683035
2016 [2017]	FPR	98	1.34093752	1.26551408	1.41706991
2016 [2017]	FPR	99	0.59147458	0.54303076	0.64417543
2016 [2017]	NPV	50	99.2303912	99.1489113	99.3080982
2016 [2017]	NPV	70	98.7528994	98.665953	98.8436059
2016 [2017]	NPV	90	97.844324	97.7465265	97.9464196
2016 [2017]	NPV	95	97.3697308	97.2651372	97.4797879
2016 [2017]	NPV	98	96.8786327	96.7604688	96.9922767
2016 [2017]	NPV	99	96.6282504	96.5045503	96.7478598
2016 [2017]	PPV	50	6.76782494	6.53280565	7.00057782
2016 [2017]	PPV	70	9.65191867	9.30048226	10.0105301
2016 [2017]	PPV	90	18.2846847	17.4677047	19.1188666
2016 [2017]	PPV	95	25.3975248	24.0574101	26.7112694
2016 [2017]	PPV	98	35.4809535	33.2924812	37.5953419
2016 [2017]	PPV	99	69.0491031	66.7743309	71.148438
2016 [2017]	TPR	50	89.7886153	88.7905911	90.7632989
2016 [2017]	TPR	70	76.835597	75.3887804	78.3212992
2016 [2017]	TPR	90	48.518691	46.7982316	50.2196098
2016 [2017]	TPR	95	33.6946707	32.1263511	35.2923905
2016 [2017]	TPR	98	18.8302554	17.5248809	20.1070239

© 2025 Kolla L et al. *JAMA Health Forum*.

2016 [2017]	TPR	99	27.5574851	26.4283368	28.6274956
2017 [2018]	Accuracy	50	53.0949122	52.7840194	53.3925345
2017 [2018]	Accuracy	70	72.0855098	71.8166213	72.3478622
2017 [2018]	Accuracy	90	89.8488202	89.6628248	90.0414696
2017 [2018]	Accuracy	95	93.6826509	93.528625	93.8317336
2017 [2018]	Accuracy	98	95.5427051	95.4141834	95.6700295
2017 [2018]	Accuracy	99	96.0068424	95.8847143	96.1241197
2017 [2018]	F1	50	12.948886	12.5313428	13.3496276
2017 [2018]	F1	70	17.6224666	17.0231037	18.1823547
2017 [2018]	F1	90	26.8914177	25.8852538	27.8869689
2017 [2018]	F1	95	28.8963114	27.5719062	30.140372
2017 [2018]	F1	98	24.2710141	22.7905046	25.6677439
2017 [2018]	F1	99	38.9309319	37.7108282	40.0834081
2017 [2018]	FPR	50	48.3880856	48.0825792	48.7069818
2017 [2018]	FPR	70	28.1065769	27.8363384	28.3839312
2017 [2018]	FPR	90	8.46103201	8.27929674	8.64161312
2017 [2018]	FPR	95	3.86534727	3.74902541	3.98893295
2017 [2018]	FPR	98	1.33767329	1.26705261	1.41143636
2017 [2018]	FPR	99	0.59366277	0.54548601	0.6421964
2017 [2018]	NPV	50	99.2054477	99.1261702	99.2818994
2017 [2018]	NPV	70	98.7141382	98.6261094	98.7990476
2017 [2018]	NPV	90	97.7567608	97.6565245	97.8565921
2017 [2018]	NPV	95	97.2608713	97.1488009	97.3673915
2017 [2018]	NPV	98	96.7636724	96.6539539	96.8723104
2017 [2018]	NPV	99	96.5084068	96.3942559	96.6188934
2017 [2018]	PPV	50	6.9776578	6.73577676	7.209359
2017 [2018]	PPV	70	9.95254006	9.58832799	10.2975811
2017 [2018]	PPV	90	18.6709714	17.8527166	19.4487715
2017 [2018]	PPV	95	25.6795662	24.3803987	26.8772819
2017 [2018]	PPV	98	35.7144133	33.7519446	37.6954674
2017 [2018]	PPV	99	69.2281026	67.1977369	71.1956581
2017 [2018]	TPR	50	89.7756722	88.8177594	90.6989888
2017 [2018]	TPR	70	76.8366203	75.4532977	78.1401746
2017 [2018]	TPR	90	48.0443693	46.4139158	49.582388
2017 [2018]	TPR	95	33.0343522	31.4660196	34.4838128
2017 [2018]	TPR	98	18.3813664	17.184487	19.5646542
2017 [2018]	TPR	99	27.0797	26.0712535	28.0417172
2018 [2019]	Accuracy	50	53.0449937	52.7037749	53.3983145
2018 [2019]	Accuracy	70	72.0074531	71.7100614	72.3059425

© 2025 Kolla L et al. *JAMA Health Forum*.

2018 [2019]	Accuracy	90	89.7971876	89.5959501	89.9897397
2018 [2019]	Accuracy	95	93.6513837	93.4874402	93.8184496
2018 [2019]	Accuracy	98	95.5292869	95.3870725	95.674477
2018 [2019]	Accuracy	99	95.9985966	95.8681537	96.1338431
2018 [2019]	F1	50	12.8529947	12.4298675	13.2707818
2018 [2019]	F1	70	17.4026184	16.814672	17.969948
2018 [2019]	F1	90	26.5578563	25.515091	27.6231644
2018 [2019]	F1	95	28.5926204	27.2713236	29.8358989
2018 [2019]	F1	98	24.1084718	22.5452041	25.659533
2018 [2019]	F1	99	38.6497914	37.3066141	40.0492708
2018 [2019]	FPR	50	48.4103051	48.0735805	48.7574333
2018 [2019]	FPR	70	28.1456582	27.8406811	28.4622237
2018 [2019]	FPR	90	8.48686153	8.30679688	8.67041438
2018 [2019]	FPR	95	3.87969628	3.74932612	4.01238239
2018 [2019]	FPR	98	1.3421221	1.26550195	1.4213086
2018 [2019]	FPR	99	0.59525672	0.54249955	0.64942293
2018 [2019]	NPV	50	99.1435703	99.0582739	99.229861
2018 [2019]	NPV	70	98.6542776	98.5611529	98.7455976
2018 [2019]	NPV	90	97.7264451	97.6209233	97.8326042
2018 [2019]	NPV	95	97.2422423	97.1249981	97.3517638
2018 [2019]	NPV	98	96.7542733	96.6339964	96.8773489
2018 [2019]	NPV	99	96.5019746	96.3738182	96.632295
2018 [2019]	PPV	50	6.92670375	6.6858674	7.1696689
2018 [2019]	PPV	70	9.8298663	9.46906102	10.1816745
2018 [2019]	PPV	90	18.4449295	17.6303535	19.2764695
2018 [2019]	PPV	95	25.4219478	24.1386585	26.6827566
2018 [2019]	PPV	98	35.5051722	33.2929575	37.9015724
2018 [2019]	PPV	99	68.9607722	66.7491958	71.3107843
2018 [2019]	TPR	50	88.9921499	87.9679437	90.0095123
2018 [2019]	TPR	70	75.7894345	74.3752603	77.2137806
2018 [2019]	TPR	90	47.4116979	45.7659681	49.0741977
2018 [2019]	TPR	95	32.6668964	31.1203955	34.1230574
2018 [2019]	TPR	98	18.2503469	16.9692612	19.5286734
2018 [2019]	TPR	99	26.8487196	25.7524534	28.0052942
2019 [2020]	Accuracy	50	52.5242004	52.2205509	52.8371038
2019 [2020]	Accuracy	70	71.7211561	71.4414636	72.0162127
2019 [2020]	Accuracy	90	90.0103824	89.8201742	90.2104061
2019 [2020]	Accuracy	95	94.0824753	93.9344993	94.2400227
2019 [2020]	Accuracy	98	96.1341069	95.765426	96.5187519

© 2025 Kolla L et al. *JAMA Health Forum*.

2019 [2020]	Accuracy	99	96.6777272	96.3454602	97.0607185
2019 [2020]	F1	50	10.6443679	10.2565128	11.0289541
2019 [2020]	F1	70	14.6639182	14.1273468	15.236211
2019 [2020]	F1	90	23.9190357	22.8665416	24.9324268
2019 [2020]	F1	95	27.2259894	25.9358268	28.5485396
2019 [2020]	F1	98	24.6531346	19.4615806	29.5223067
2019 [2020]	F1	99	39.8184372	35.1002277	44.0090607
2019 [2020]	FPR	50	48.6971449	48.3724129	48.9988607
2019 [2020]	FPR	70	28.4687297	28.16863	28.7670801
2019 [2020]	FPR	90	8.70117453	8.51647213	8.88145618
2019 [2020]	FPR	95	4.0190846	3.88938425	4.14612503
2019 [2020]	FPR	98	1.41132641	1.18386888	1.6567523
2019 [2020]	FPR	99	0.62698967	0.47481677	0.79743713
2019 [2020]	NPV	50	99.3930707	99.3195156	99.4635652
2019 [2020]	NPV	70	98.9977146	98.9197465	99.0736345
2019 [2020]	NPV	90	98.2656907	98.1786276	98.3564941
2019 [2020]	NPV	95	97.8691689	97.7772912	97.9666106
2019 [2020]	NPV	98	97.4502574	97.1390816	97.7897167
2019 [2020]	NPV	99	97.2336907	96.9107746	97.5881723
2019 [2020]	PPV	50	5.65548086	5.441387	5.87101704
2019 [2020]	PPV	70	8.09704397	7.78139346	8.44259009
2019 [2020]	PPV	90	15.7046538	14.9462782	16.4643901
2019 [2020]	PPV	95	22.1377457	20.9631798	23.3383412
2019 [2020]	PPV	98	31.629096	25	38.1228094
2019 [2020]	PPV	99	64.5707137	57.0468617	71.8413491
2019 [2020]	TPR	50	90.3084027	89.2075449	91.3498576
2019 [2020]	TPR	70	77.595566	76.0069386	79.1175094
2019 [2020]	TPR	90	50.1504056	48.2283589	51.9417813
2019 [2020]	TPR	95	35.3512983	33.6863054	37.0755371
2019 [2020]	TPR	98	20.1982956	15.6146179	24.7719435
2019 [2020]	TPR	99	28.7843578	24.778695	32.5142525
2020 [2021]	Accuracy	50	52.3094157	51.9915477	52.6303073
2020 [2021]	Accuracy	70	71.4894565	71.2024378	71.8014403
2020 [2021]	Accuracy	90	89.880651	89.6889603	90.0708525
2020 [2021]	Accuracy	95	94.0537388	93.904388	94.2142259
2020 [2021]	Accuracy	98	96.2136837	95.8087464	96.5956567
2020 [2021]	Accuracy	99	96.7815747	96.415722	97.1302011
2020 [2021]	F1	50	9.96746413	9.59644662	10.3287155
2020 [2021]	F1	70	13.5387546	13.0458828	14.0695733

© 2025 Kolla L et al. *JAMA Health Forum*.

2020 [2021]	F1	90	22.0041058	21.0708694	22.9805447
2020 [2021]	F1	95	25.431907	24.1211942	26.7070923
2020 [2021]	F1	98	23.8894626	18.6950913	28.9990622
2020 [2021]	F1	99	38.502352	33.623349	42.9069694
2020 [2021]	FPR	50	48.8076792	48.4806361	49.1215768
2020 [2021]	FPR	70	28.6193977	28.3007575	28.8990788
2020 [2021]	FPR	90	8.8349092	8.65127731	9.02118963
2020 [2021]	FPR	95	4.10777979	3.97399194	4.23382242
2020 [2021]	FPR	98	1.44896078	1.20088675	1.70712191
2020 [2021]	FPR	99	0.65166433	0.48396173	0.83438808
2020 [2021]	NPV	50	99.3305037	99.2557737	99.4020249
2020 [2021]	NPV	70	98.9393088	98.8628349	99.0147915
2020 [2021]	NPV	90	98.2808424	98.1943907	98.370544
2020 [2021]	NPV	95	97.9361408	97.842355	98.030502
2020 [2021]	NPV	98	97.5709598	97.2397203	97.8718781
2020 [2021]	NPV	99	97.3667213	97.0249812	97.6772669
2020 [2021]	PPV	50	5.28025758	5.07613705	5.48264855
2020 [2021]	PPV	70	7.44059566	7.15049475	7.75380993
2020 [2021]	PPV	90	14.2749651	13.617085	14.9873639
2020 [2021]	PPV	95	20.2817486	19.1398064	21.3987218
2020 [2021]	PPV	98	29.7098875	23.1568368	36.4217593
2020 [2021]	PPV	99	61.5935373	53.703393	69.2772069
2020 [2021]	TPR	50	88.7457818	87.5637956	89.8743505
2020 [2021]	TPR	70	75.0399618	73.4578805	76.6005424
2020 [2021]	TPR	90	47.9859873	46.161879	49.8719403
2020 [2021]	TPR	95	34.0878608	32.265045	35.8563656
2020 [2021]	TPR	98	19.9759923	15.4404854	24.7460112
2020 [2021]	TPR	99	28.0038238	24	31.7904774

Note *: Year is notated as: Year of Risk Score Calculation [Year of Outcome Measurement]
**Metrics are abbreviated as: TPR (True Positive Rate); FPR (False Positive Rate); PPV (Positive Predictive Value); NPV (Negative Predictive Value)

eTable 5: Performance drift, measured by absolute change in metric (%), separated by time period, metric, and threshold, with 95% confidence intervals

Period*	Metric**	Threshold	Absolute Drift (%)	Lower_Bound	Upper_Bound
2016-2018 [2017-2019]	Accuracy	50	0.04362526	-0.2994146	0.4180859
2016-2018 [2017-2019]	Accuracy	70	-0.0148952	-0.3326203	0.32755517
2016-2018 [2017-2019]	Accuracy	90	-0.0912052	-0.3272268	0.15028416
2016-2018 [2017-2019]	Accuracy	95	-0.119932	-0.3252005	0.08061591
2016-2018 [2017-2019]	Accuracy	98	-0.121371	-0.3010637	0.05330501
2016-2018 [2017-2019]	Accuracy	99	-0.1266968	-0.2990522	0.03920258
2016-2018 [2017-2019]	F1	50	0.26608432	-0.3271122	0.85425428
2016-2018 [2017-2019]	F1	70	0.25306962	-0.5983737	1.05519033
2016-2018 [2017-2019]	F1	90	-0.0021478	-1.4641654	1.4819376
2016-2018 [2017-2019]	F1	95	-0.3709755	-2.2409235	1.40655703
2016-2018 [2017-2019]	F1	98	-0.4947521	-2.7414884	1.73378345
2016-2018 [2017-2019]	F1	99	-0.7433736	-2.4941603	1.18523451
2016-2018 [2017-2019]	FPR	50	-0.0289714	-0.3843618	0.31114993
2016-2018 [2017-2019]	FPR	70	-0.0204877	-0.3634911	0.29383077

© 2025 Kolla L et al. *JAMA Health Forum*.

2016-2018 [2017-2019]	FPR	90	-0.0046447	-0.2192157	0.2042177
2016-2018 [2017-2019]	FPR	95	0.0037055	-0.1566731	0.1700803
2016-2018 [2017-2019]	FPR	98	0.00118458	-0.0980657	0.09977629
2016-2018 [2017-2019]	FPR	99	0.00378214	-0.0662183	0.06945664
2016-2018 [2017-2019]	NPV	50	-0.086821	-0.2008509	0.03443149
2016-2018 [2017-2019]	NPV	70	-0.0986218	-0.2238434	0.03270264
2016-2018 [2017-2019]	NPV	90	-0.1178789	-0.2653395	0.02818126
2016-2018 [2017-2019]	NPV	95	-0.1274885	-0.283261	0.03542589
2016-2018 [2017-2019]	NPV	98	-0.1243594	-0.2953865	0.04020702
2016-2018 [2017-2019]	NPV	99	-0.1262758	-0.2963549	0.03467525
2016-2018 [2017-2019]	PPV	50	0.15887882	-0.1811146	0.49513039
2016-2018 [2017-2019]	PPV	70	0.17794764	-0.3451363	0.67144188
2016-2018 [2017-2019]	PPV	90	0.16024476	-0.979099	1.35202339
2016-2018 [2017-2019]	PPV	95	0.02442305	-1.7825235	1.78884133
2016-2018 [2017-2019]	PPV	98	0.02421871	-2.9797915	3.3438247

© 2025 Kolla L et al. *JAMA Health Forum*.

2016-2018 [2017-2019]	PPV	99	-0.0883309	-2.9810024	3.23778152
2016-2018 [2017-2019]	TPR	50	-0.7964654	-2.2273113	0.67034943
2016-2018 [2017-2019]	TPR	70	-1.0461626	-3.0992411	0.95808399
2016-2018 [2017-2019]	TPR	90	-1.1069931	-3.4221695	1.10910137
2016-2018 [2017-2019]	TPR	95	-1.0277743	-3.280351	1.0374145
2016-2018 [2017-2019]	TPR	98	-0.5799085	-2.4003861	1.26009491
2016-2018 [2017-2019]	TPR	99	-0.7087656	-2.1708444	0.88906929
**2016-2020 [2017-2021]**	**Accuracy**	**50**	**-0.6919527**	**-1.0881589**	**-0.2885209**
**2016-2020 [2017-2021]**	**Accuracy**	**70**	**-0.5328918**	**-0.9036202**	**-0.1595287**
2016-2020 [2017-2021]	Accuracy	90	-0.0077418	-0.2693358	0.26066725
**2016-2020 [2017-2021]**	**Accuracy**	**95**	**0.28242307**	**0.06532845**	**0.50094592**
**2016-2020 [2017-2021]**	**Accuracy**	**98**	**0.56302587**	**0.00030151**	**1.16533607**
**2016-2020 [2017-2021]**	**Accuracy**	**99**	**0.65628136**	**0.12565653**	**1.19690533**
**2016-2020 [2017-2021]**	**F1**	**50**	**-2.6194462**	**-3.1563988**	**-2.0394788**
**2016-2020 [2017-2021]**	**F1**	**70**	**-3.6107941**	**-4.4265651**	**-2.7883008**

© 2025 Kolla L et al. *JAMA Health Forum*.

**2016-2020 [2017-2021]**	**F1**	**90**	**-4.5558983**	**-6.0923265**	**-3.0338773**
**2016-2020 [2017-2021]**	**F1**	**95**	**-3.5316889**	**-5.5507512**	**-1.5373788**
2016-2020 [2017-2021]	F1	98	-0.7137613	-8.1779472	6.70175631
2016-2020 [2017-2021]	F1	99	-0.8908129	-7.6812152	5.70970787
2016-2020 [2017-2021]	FPR	50	0.36840275	-0.0308921	0.74667672
**2016-2020 [2017-2021]**	**FPR**	**70**	**0.45325179**	**0.08103679**	**0.83403945**
**2016-2020 [2017-2021]**	**FPR**	**90**	**0.34340296**	**0.10237209**	**0.58377537**
**2016-2020 [2017-2021]**	**FPR**	**95**	**0.23178901**	**0.04285704**	**0.40206572**
2016-2020 [2017-2021]	FPR	98	0.10802327	-0.238395	0.4649964
2016-2020 [2017-2021]	FPR	99	0.06018974	-0.188493	0.31010661
2016-2020 [2017-2021]	NPV	50	0.10011247	-0.0106472	0.21726733
**2016-2020 [2017-2021]**	**NPV**	**70**	**0.1864094**	**0.06400708**	**0.30818622**
**2016-2020 [2017-2021]**	**NPV**	**90**	**0.43651836**	**0.30137764**	**0.57271702**
**2016-2020 [2017-2021]**	**NPV**	**95**	**0.56641006**	**0.41869058**	**0.70926634**
**2016-2020 [2017-2021]**	**NPV**	**98**	**0.69232704**	**0.20595178**	**1.19166334**

© 2025 Kolla L et al. *JAMA Health Forum*.

**2016-2020 [2017-2021]**	**NPV**	**99**	**0.73847085**	**0.23032243**	**1.24436888**
**2016-2020 [2017-2021]**	**PPV**	**50**	**-1.4875674**	**-1.7907048**	**-1.1576502**
**2016-2020 [2017-2021]**	**PPV**	**70**	**-2.211323**	**-2.698612**	**-1.7105298**
**2016-2020 [2017-2021]**	**PPV**	**90**	**-4.0097197**	**-5.1470708**	**-2.8278493**
**2016-2020 [2017-2021]**	**PPV**	**95**	**-5.1157761**	**-7.0026616**	**-3.2481017**
2016-2020 [2017-2021]	PPV	98	-5.771066	-15.898296	4.27280923
2016-2020 [2017-2021]	PPV	99	-7.4555657	-18.732193	3.92422912
2016-2020 [2017-2021]	TPR	50	-1.0428334	-2.6721691	0.5720079
2016-2020 [2017-2021]	TPR	70	-1.7956353	-4.0967334	0.43447662
2016-2020 [2017-2021]	TPR	90	-0.5327037	-3.1751519	2.16804734
2016-2020 [2017-2021]	TPR	95	0.3931901	-2.2607102	2.94754151
2016-2020 [2017-2021]	TPR	98	1.14573692	-5.335293	7.82723317
2016-2020 [2017-2021]	TPR	99	0.44633869	-5.0961421	5.80205143
2019-2020 [2020-2021]	Accuracy	50	-0.2147846	-0.5460181	0.08367393
2019-2020 [2020-2021]	Accuracy	70	-0.2316996	-0.4949104	0.03515359

© 2025 Kolla L et al. *JAMA Health Forum*.

2019-2020 [2020-2021]	Accuracy	90	-0.1297314	-0.3380152	0.08019173
2019-2020 [2020-2021]	Accuracy	95	-0.0287364	-0.1985555	0.155348
2019-2020 [2020-2021]	Accuracy	98	0.0795768	-0.416881	0.57350195
2019-2020 [2020-2021]	Accuracy	99	0.1038475	-0.3727793	0.55449336
**2019-2020 [2020-2021]**	**F1**	**50**	**-0.6769038**	**-1.1751593**	**-0.1829607**
**2019-2020 [2020-2021]**	**F1**	**70**	**-1.1251636**	**-1.8359177**	**-0.4201421**
**2019-2020 [2020-2021]**	**F1**	**90**	**-1.9149299**	**-3.2324939**	**-0.5730716**
**2019-2020 [2020-2021]**	**F1**	**95**	**-1.7940824**	**-3.5905282**	**-0.0558528**
2019-2020 [2020-2021]	F1	98	-0.763672	-8.0437498	6.50066728
2019-2020 [2020-2021]	F1	99	-1.3160853	-7.6255221	4.71318708
2019-2020 [2020-2021]	FPR	50	0.11053431	-0.1781778	0.42279841
2019-2020 [2020-2021]	FPR	70	0.15066802	-0.1184445	0.39974387
2019-2020 [2020-2021]	FPR	90	0.13373467	-0.0542803	0.31958061
2019-2020 [2020-2021]	FPR	95	0.08869519	-0.0601458	0.23068325
2019-2020 [2020-2021]	FPR	98	0.03763438	-0.2742907	0.35846703

© 2025 Kolla L et al. *JAMA Health Forum*.

2019-2020 [2020-2021]	FPR	99	0.02467466	-0.1879444	0.2448861
2019-2020 [2020-2021]	NPV	50	-0.062567	-0.16422	0.0443239
2019-2020 [2020-2021]	NPV	70	-0.0584057	-0.1678135	0.0489571
2019-2020 [2020-2021]	NPV	90	0.01515166	-0.1117724	0.14137159
2019-2020 [2020-2021]	NPV	95	0.06697191	-0.0593229	0.19958379
2019-2020 [2020-2021]	NPV	98	0.12070235	-0.3404606	0.55507338
2019-2020 [2020-2021]	NPV	99	0.13303061	-0.3356979	0.57515917
**2019-2020 [2020-2021]**	**PPV**	**50**	**-0.3752233**	**-0.6548889**	**-0.0970715**
**2019-2020 [2020-2021]**	**PPV**	**70**	**-0.6564483**	**-1.0796844**	**-0.2410942**
**2019-2020 [2020-2021]**	**PPV**	**90**	**-1.4296888**	**-2.3888202**	**-0.4628383**
**2019-2020 [2020-2021]**	**PPV**	**95**	**-1.855997**	**-3.4432055**	**-0.299588**
2019-2020 [2020-2021]	PPV	98	-1.9192085	-11.152699	7.57500489
2019-2020 [2020-2021]	PPV	99	-2.9771763	-13.400883	7.94302332
2019-2020 [2020-2021]	TPR	50	-1.5626209	-3.1362132	0.05976011
**2019-2020 [2020-2021]**	**TPR**	**70**	**-2.5556042**	**-4.8115808**	**-0.4035239**

© 2025 Kolla L et al. *JAMA Health Forum*.

2019-2020 [2020-2021]	TPR	90	-2.1644182	-4.7649252	0.29780614
2019-2020 [2020-2021]	TPR	95	-1.2634375	-3.6567088	0.9606897
2019-2020 [2020-2021]	TPR	98	-0.2223033	-6.5533608	6.30866691
2019-2020 [2020-2021]	TPR	99	-0.780534	-6.039658	4.2043765

*Note: Period is separated into three levels: 2016–2018 [2017–2019], 2019–2020 [2020–2021], and 2016–2020 [2017–2021].
**Metrics are abbreviated as: TPR (True Positive Rate); FPR (False Positive Rate); PPV (Positive Predictive Value); NPV (Negative Predictive Value)
Caption: Bolded values represent drift measures with 95% CI that do not cross zero in the respective time-period, metric, and threshold.

eFigure 2: Changes in the number of false positives identified due to drift, grouped by time period and threshold.

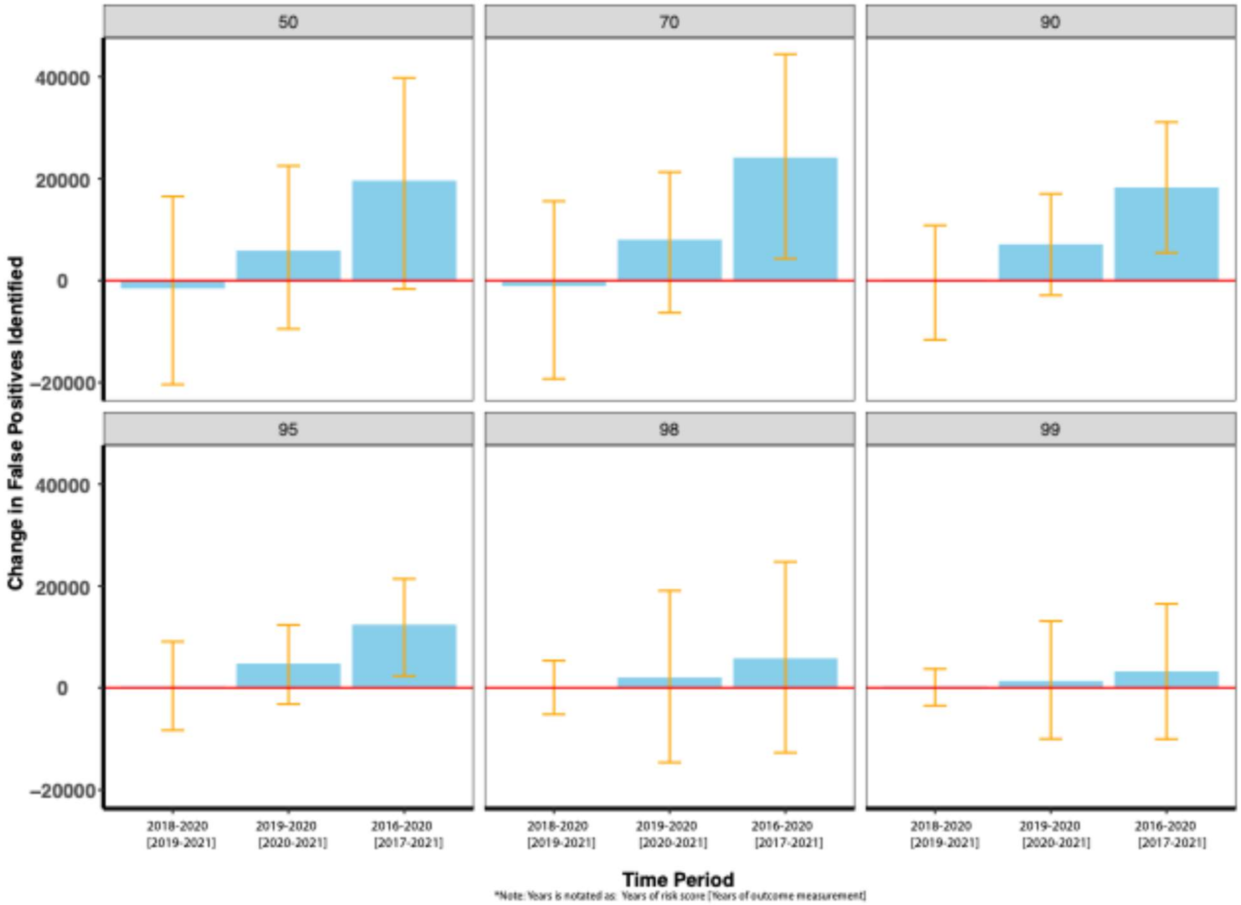

© 2025 Kolla L et al. *JAMA Health Forum*.

Caption: The figure illustrates the absolute change in the controls identified due to drift across four risk thresholds (50, 70, 90, 95, 98, 99) over three time periods: 2016–2018 [2017–2019], 2019–2020 [2020–2021], and 2016–2020 [2017–2021]. The x-axis represents time period, y-axis displays absolute change in false positives, and each panel corresponds to a different risk threshold. 95% confidence intervals are shown by the orange lines surrounding each point estimate. See **Methods** for description on how 95% bootstrapped confidence intervals were obtained. The red horizontal line at zero represents no change. *Calculation for change in false positives identified = (% Drift in FPR in time period) * (#Controls in the final year of time period)*

Performance drift can change the number of false positives identified among all controls, those who do not experience the 90-day combined outcome. We measured the number of individuals impacted by drift by multiplying the absolute change (i.e. drift) in false positive rates within a given time-period by the number of controls in the final year of the time-period. For example, change in the number of false positives identified in the 2016-2018 [2017-2019] time-period is equal to the absolute change in FPR between 2016[2017] and 2018[2019], multiplied by the number of controls observed in 2021.

eFigure 3: Changes in the number of true positives identified due to drift, grouped by time period and threshold.

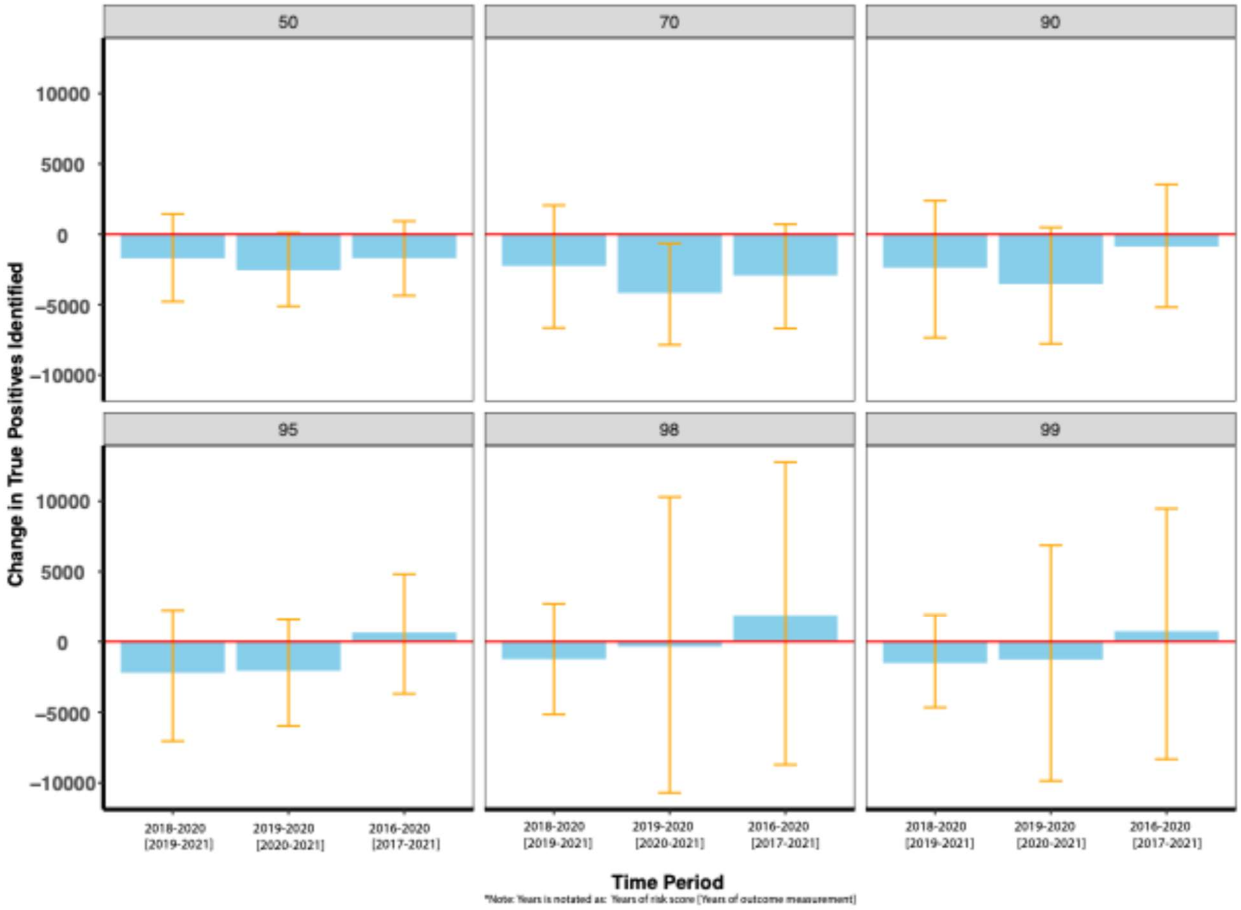

© 2025 Kolla L et al. *JAMA Health Forum*.

Caption: The figure illustrates the absolute change in the cases identified due to drift across four risk thresholds (50, 70, 90, 95, 98, 99) over three time periods: 2016–2018 [2017–2019], 2019–2020 [2020–2021], and 2016–2020 [2017–2021]. The x-axis represents time period, y-axis displays absolute change in true positives, and each panel corresponds to a different risk threshold. 95% confidence intervals are shown by the orange lines surrounding each point estimate. See **Methods** for description on how 95% bootstrapped confidence intervals were obtained. The red horizontal line at zero represents no change. *Calculation for change in true positives identified = (% Drift in TPR in time period) * (#Cases in the final year of time period)*

Performance drift can change the number of true positives, identified by the algorithm, among all cases, those who experience the 90-day combined outcome. We measured the number of individuals impacted by drift by multiplying the absolute change (i.e. drift) in true positive rates within a given time-period by the number of cases in the final year of the time-period. For example, change in the number of true positives identified in the 2016-2018 [2017-2019] time-period is equal to the absolute change in TPR between 2016[2017] and 2018[2019], multiplied by the number of cases observed in 2021.

eTable 6: Classification rates, by year, metric, and threshold (50th, 70th, 95th, 98th, 99th), with 95% confidence intervals

Year*	Metric**	Threshold	Classification Percentage(%)	Lower Bound	Upper Bound
2016	TP	50	3.38374035	3.36874274	3.39873796
2017	TP	50	3.48856664	3.47336971	3.50376356
2018	TP	50	3.46260335	3.44735089	3.47785581
2019	TP	50	2.82773841	2.81400292	2.84147389
2020	TP	50	2.63990225	2.62649027	2.65331422
2016	TP	70	2.89559773	2.88168901	2.90950644
2017	TP	70	2.98577179	2.97167602	2.99986757
2018	TP	70	2.94889774	2.93478469	2.96301079
2019	TP	70	2.42967383	2.41691572	2.44243193
2020	TP	70	2.23219808	2.21983939	2.24455678
2016	TP	95	1.26980483	1.26051747	1.27909218
2017	TP	95	1.28367225	1.27434906	1.29299543
2018	TP	95	1.27103913	1.26169385	1.28038441
2019	TP	95	1.10692052	1.09825101	1.11559003
2020	TP	95	1.01400448	1.00562311	1.02238585
2016	TP	98	0.70963	0.70266746	0.71659254
2017	TP	98	0.71427615	0.70730155	0.72125075
2018	TP	98	0.71010434	0.70309941	0.71710928
2019	TP	98	0.63244941	0.62588058	0.63901825
2020	TP	98	0.59422167	0.58779201	0.60065134

© 2025 Kolla L et al. *JAMA Health Forum*.

2016	TP	99	1.26980483	1.26051747	1.27909218
2017	TP	99	1.28367225	1.27434906	1.29299543
2018	TP	99	1.27103913	1.26169385	1.28038441
2019	TP	99	1.10692052	1.09825101	1.11559003
2020	TP	99	1.01400448	1.00562311	1.02238585
2016	FP	50	46.6137165	46.5723384	46.6550945
2017	FP	50	46.5076748	46.4663652	46.5489844
2018	FP	50	46.5265903	46.4849791	46.5682015
2019	FP	50	47.1722258	47.1308614	47.2135903
2020	FP	50	47.3558074	47.3140368	47.3975779
2016	FP	70	27.1046315	27.0677618	27.1415013
2017	FP	70	27.0143264	26.9775509	27.0511019
2018	FP	70	27.05047	27.0134114	27.0875286
2019	FP	70	27.5772501	27.5402191	27.6142812
2020	FP	70	27.7680624	27.7305957	27.8055292
2016	FP	95	3.72991399	3.71419613	3.74563186
2017	FP	95	3.71513589	3.69947166	3.73080013
2018	FP	95	3.7287317	3.71292578	3.74453761
2019	FP	95	3.89322961	3.8772014	3.90925783
2020	FP	95	3.98558652	3.96922127	4.00195177
2016	FP	98	1.2904008	1.28103941	1.2997622
2017	FP	98	1.28568993	1.27635951	1.29502034
2018	FP	98	1.28989819	1.28048473	1.29931165
2019	FP	98	1.36713165	1.35750956	1.37675373
2020	FP	98	1.40585885	1.39600957	1.41570814
2016	FP	99	0.56918333	0.56294332	0.57542333
2017	FP	99	0.57059242	0.56435416	0.57683068
2018	FP	99	0.57209442	0.56580257	0.57838628
2019	FP	99	0.6073559	0.60091789	0.61379392
2020	FP	99	0.63227941	0.62564831	0.63891051
2016	TN	50	49.6175237	49.5760516	49.6589958
2017	TN	50	49.6062223	49.5648129	49.6476318
2018	TN	50	49.5822655	49.5405549	49.623976
2019	TN	50	49.6963399	49.6549099	49.7377699
2020	TN	50	49.6695135	49.6276853	49.7113417
2016	TN	70	69.1266087	69.0882898	69.1649276
2017	TN	70	69.0995707	69.0613003	69.137841
2018	TN	70	69.0583857	69.0198228	69.0969487
2019	TN	70	69.2913156	69.2530928	69.3295385

© 2025 Kolla L et al. *JAMA Health Forum*.

2020	TN	70	69.2572584	69.2186562	69.2958607
2016	TN	95	92.5013262	92.4794806	92.5231718
2017	TN	95	92.3987612	92.376812	92.4207104
2018	TN	95	92.380124	92.3579903	92.4022577
2019	TN	95	92.9753362	92.9541599	92.9965124
2020	TN	95	93.0397344	93.0184453	93.0610234
2016	TN	98	94.9408394	94.9226606	94.9590181
2017	TN	98	94.8282071	94.8098658	94.8465485
2018	TN	98	94.8189575	94.8004671	94.837448
2019	TN	98	95.5014341	95.4842592	95.5186091
2020	TN	98	95.619462	95.6023404	95.6365837
2016	TN	99	95.6620569	95.6451598	95.6789539
2017	TN	99	95.5433047	95.5262144	95.5603949
2018	TN	99	95.5367613	95.5195347	95.553988
2019	TN	99	96.2612099	96.2454902	96.2769296
2020	TN	99	96.3930415	96.3774423	96.4086406
2016	FN	50	0.38482245	0.37968684	0.38995805
2017	FN	50	0.39730417	0.39209414	0.40251419
2018	FN	50	0.4283054	0.42285742	0.43375338
2019	FN	50	0.30346348	0.29890577	0.30802118
2020	FN	50	0.33477688	0.32994454	0.33960922
2016	FN	70	0.87296507	0.86524906	0.88068108
2017	FN	70	0.90009901	0.89227689	0.90792113
2018	FN	70	0.94201101	0.93395234	0.95006969
2019	FN	70	0.70152806	0.69461219	0.70844393
2020	FN	70	0.74248105	0.73529926	0.74966284
2016	FN	95	2.49875797	2.48581108	2.51170487
2017	FN	95	2.60219856	2.58901333	2.61538378
2018	FN	95	2.61986962	2.60654467	2.63319458
2019	FN	95	2.02428137	2.01261198	2.03595075
2020	FN	95	1.96067465	1.9490759	1.9722734
2016	FN	98	3.0589328	3.04464921	3.07321638
2017	FN	98	3.17159465	3.15708079	3.18610852
2018	FN	98	3.18080441	3.16616445	3.19544437
2019	FN	98	2.49875247	2.48581886	2.51168609
2020	FN	98	2.38045745	2.36770461	2.39321029
2016	FN	99	3.33803519	3.32313569	3.35293468
2017	FN	99	3.4566766	3.44154679	3.4718064
2018	FN	99	3.46303814	3.44778476	3.47829153

© 2025 Kolla L et al. *JAMA Health Forum*.

2019	FN	99	2.73864215	2.72511859	2.75216571
2020	FN	99	2.6069456	2.59361535	2.62027585
2016	Prevalence		3.7685628	3.75276688	3.78435871
2017	Prevalence		3.8858708	3.86986488	3.90187672
2018	Prevalence		3.89090875	3.87477637	3.90704114
2019	Prevalence		3.13120189	3.11677074	3.14563303
2020	Prevalence		2.97467913	2.96046661	2.98889164

Note *: Year is notated as: Year of Risk Score Calculation [Year of Outcome Measurement]
**: TP = True Positive; FP = False Positive; FN = False Negative; TN = True Negative

eTable 7: Possible covariate drivers of drift, with corresponding variable description, standardized mean difference (SMD) and variable ID for each drift time period.

**Variable**	**Description**	**2016-2020 [2017-2021] SMD**	**2016-2018 [2017-2019] SMD**	**2019-2020 [2020-2021] SMD**	**Type**	**2019-2020 [2020-2021]**	**2016-2020 [2017-2021]**	**2016-2018 [2017-2019]**
agegrp_c1	Age < 55 vs Age >= 85	0.0412	0.0149	0.013	Demographic	1	1	1
agegrp_c2	Age 55-64 vs Age >= 85	-0.0284	-0.0153	-0.007	Demographic	2	2	2
agegrp_c3	Age 65-74 vs Age >= 85	-0.0445	-0.005	-0.022	Demographic	3	3	3
albuminc0	Most Recent Albumin Lab result prior 1 year: > 3.4 vs No Albumin Lab	-0.1141	0.0667	-0.211	Laboratory	5	5	
bunc0	Most Recent Blood, Urine, Nitrogen Lab result prior 1 year: 0-16 vs No Lab	-0.1141	0.0237	-0.137	Laboratory	9	9	

© 2025 Kolla L et al. *JAMA Health Forum*.

bunc1	Most Recent Blood, Urine, Nitrogen Lab result prior 1 year: 17-25 vs No Lab	-0.0754	0.0224	-0.102	Laboratory	10		
cpt21to30fon730_c0	Phone CPTs 21to30 mins prior 2 years: 0 vs > 1	-0.3189	-0.0508	-0.241	Utilization	13	13	
cpt21to30fon730_c1	Phone CPTs 21to30 mins prior 2 years: 1 vs > 1	0.1366	0.0263	0.0936	Utilization		14	
cptoffvisest90_c0	Established Office Visit CPTs prior 90 days: 0 vs > 4	0.2226	-0.0471	0.2765	Utilization	17	17	
cptoffvisest90_c1	Established Office Visit CPTs prior 90 days: 1-4 vs > 4	-0.1766	0.048	-0.232	Utilization	18	18	
diastc2	Most Recent Diastolic Blood Pressure Measurement prior 1 year: 60 < diast < 90 or Unknown vs >= 90	0.1009	0.0263	0.0568	Laboratory		24	
hospbedc0	Hosp Stays & Bed Days of Care prior 1 year: level 0 vs 4	0.0513	0.0018	0.0451	Utilization	34	34	34
i_admtall365_c0	All Hospital Admissions prior 1 year: 0 vs > 2	0.0618	0.0025	0.0544	Utilization	38	38	38
prior1245_0	Vet Priority level 0 vs 5	-0.1486	-0.0682	-0.038	Demographic		55	
prior1245_1	Vet Priority level 1 vs 5	0.1732	0.0742	0.0494	Demographic		56	
pulsec1	Most Recent Pulse Vital Measurement prior 1 year: < 60 vs >= 90	-0.1484	-0.0131	-0.136	Laboratory	59	59	

© 2025 Kolla L et al. *JAMA Health Forum*.

pulsec2	Most Recent Pulse Vital Measurement prior 1 year: 60 <= pulse < 90 or Unknown vs >= 90	0.14	-0.0046	0.1429	Laboratory	60	60	
respc1	Most Recent Respiration Vital Measurement prior 1 year: < 18 vs >= 20	-0.1384	0.0555	-0.215	Laboratory	61	61	
respc2	Most Recent Respiration Vital Measurement prior 1 year: 18 <= respir < 20 or Unknown vs >= 20	0.268	-0.018	0.2793	Laboratory	62	62	
systc1	Most Recent Systolic Blood Pressure Measurement prior 1 year: < 110 vs >= 160	-0.0858	0.0138	-0.109	Laboratory	75		
systc2	Most Recent Systolic Blood Pressure Measurement prior 1 year: 110 <= syst < 140 or Unknown vs >= 160	0.1088	-0.0106	0.1103	Laboratory	76	76	
visits_c0	Outpatient Visits prior 3 years: 0-90 vs > 180	-0.1308	-0.0779	-0.024	Utilization		78	

© 2025 Kolla L et al. *JAMA Health Forum*.

eFigure 4: Covariate Shift in VA CAN covariates between 2019-2020, grouped by covariate category

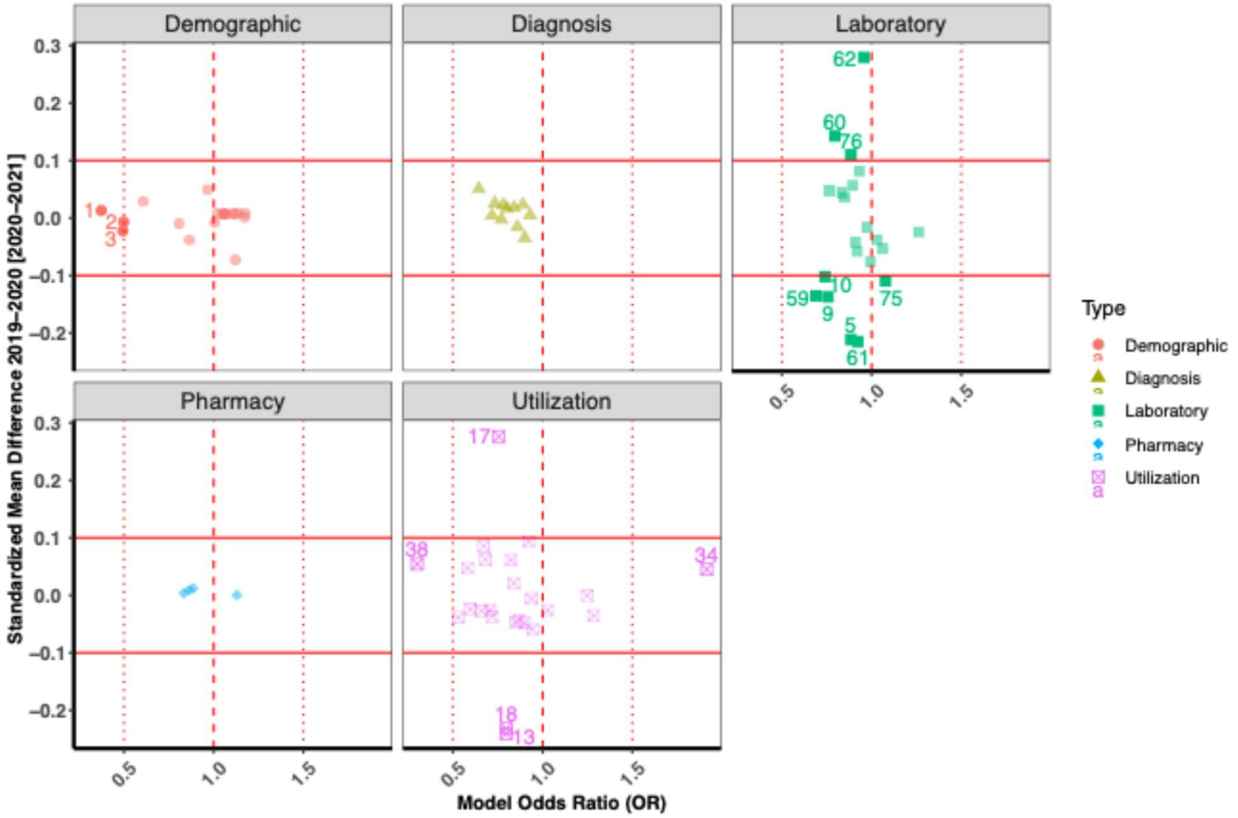

Caption: Covariate shift in VA CAN score predictors between 2019–2020, grouped by covariate category. The y-axis displays standardized mean differences (SMD), a measure of covariate shift, in covariate distributions between 2019 and 2020. The x-axis displays algorithm odds ratios. Each point is a different covariate, and covariates are grouped into five categories: Demographics, Diagnoses, Laboratory, Pharmacy, and Utilization. The vertical red dashed line delineates where the algorithm odds ratio is 1, indicating no predictive value of covariates that fall on the line. Covariates outside of the horizontal solid red lines, which represent thresholds for significant covariate shift, and covariates outside of the vertical dotted red lines, which represent thresholds for significant predictive effect, are potential drivers of drift and labeled by

© 2025 Kolla L et al. *JAMA Health Forum*.

their variable ID. Original algorithm odds ratios are in **eTable 1.** All variable IDs and corresponding SMD, OR, and variable description are in **eTable 7.** The main drivers are related to hospital utilization patterns (e.g. office visits vs. telehealth visits, hospital admissions, hospital stays and bed days of care, and outpatient visits); and laboratory markers (e.g. respiration and pulse vitals, BUN, albumin, blood pressures).

eFigure 5: Covariate Shift in VA CAN covariates between 2016-2018, grouped by covariate category

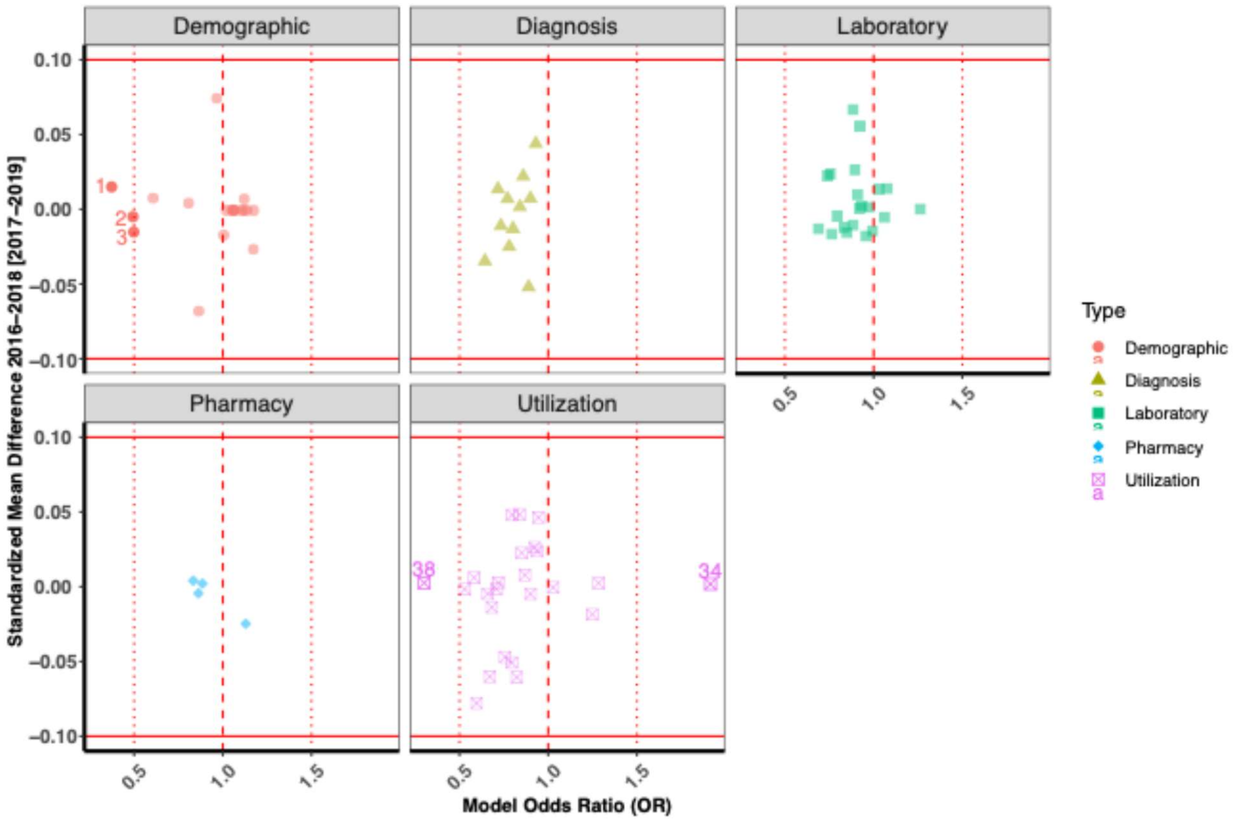

Caption: Covariate shift in VA CAN score predictors between 2016–2018, grouped by covariate category. The y-axis displays standardized mean differences (SMD), a measure of covariate shift, in covariate distributions between 2016 and 2018. The x-axis displays algorithm odds ratios. Each point is a different covariate, and covariates are grouped into five categories: Demographics, Diagnoses, Laboratory, Pharmacy, and Utilization. The vertical red dashed line delineates where the algorithm odds ratio is 1, indicating no predictive value of covariates that

© 2025 Kolla L et al. *JAMA Health Forum*.

fall on the line. Covariates outside of the horizontal solid red lines, which represent thresholds for significant covariate shift, and covariates outside of the vertical dotted red lines, which represent thresholds for significant predictive effect, are potential drivers of drift and labeled by their variable ID. Original algorithm odds ratios are in **eTable 1.** All variable IDs and corresponding SMD, OR, and variable description are in **eTable 7.**

eTable 8: Algorithm retraining, recalculated absolute drift (%) in 2016-2020 [2017-2021]* time period, 90th risk percentile threshold

Validation	TPR	FPR	PPV	NPV	Accuracy	F1score
No Drivers	**-1.30**	0.35	-4.09	0.42	**-0.02**	-4.74
No Demographics Drivers	**-1.58**	0.37	-4.32	0.40	**-0.07**	-5.04
No Laboratory Drivers	**-1.22**	0.37	-4.26	0.40	**-0.05**	-4.94
No Utilization Drivers	**-1.22**	0.36	-4.19	0.41	**-0.04**	-4.85
Recalibrated with 2018 data	-0.43	0.34	-3.99	0.43	-0.005	-4.52
Recalibrated with 2019 data	-0.26	0.34	-3.97	0.44	**-0.0005**	-4.47
Original**	-0.53	0.34	-4.00	0.43	-0.007	-4.55

*Note: Years notated as: Years of Risk Score [Year of Outcome Measurement]

** Original drift measurements at the 90th percentile, reported in Figure 1 of the main manuscript

Caption: Table shows recalculated drift for each driver validation or recalibration method, compared against original algorithm drift measures in the final row. Bolded values represent validation method and metrics for which a deviation of measured drift from original measured drift was noted.

© 2025 Kolla L et al. *JAMA Health Forum*.

eTable 9: Clinical Impact Analysis, 98th and 99th percentile risk thresholds

	Year*	Percentage of high-risk patients who receive palliative care (i.e. quality metric)	False positive rate among high-risk Veterans	False positive rate among high-risk Veterans who received palliative care
**98th Percentile**	2018 [2019]	10.75 [10.56 – 10.93]	64.49 [64.21 – 64.77]	54.56 [53.67 – 55.46]
	2019 [2020]	8.96 [8.79 – 9.12]	68.37 [68.09 – 68.64]	57.07 [56.10 – 58.04]
	2020 [2021]	10.28 [10.10 – 10.46]	70.29 [70.01 – 70.56]	63.09 [62.20 – 63.98]
**99th Percentile**	2018 [2019]	6.90 [6.75 – 7.06]	31.03 [30.75 – 31.32]	48.14 [46.97 – 49.31]
	2019 [2020]	6.33 [6.17 – 6.48]	35.42 [35.12 – 35.73]	52.27 [51.02 – 53.53]
	2020 [2021]	7.44 [7.27 – 7.61]	38.40 [38.08 – 38.72]	57.61 [56.43 – 58.79]

*Year is notated as: Fiscal Year / Year of Risk Score Calculation [Year of Outcome Measurement]. Fiscal years run from October of the specified year to September of the following year. Palliative care referrals are binned by fiscal year.

Caption: Table summarizes the impact of drift on quality metrics calculated across three fiscal years. Presented are values of a VA quality metric and proportions of false positives among those flagged as "high-risk" at the 90th percentile risk threshold as well as those within the "high-risk" group who received a palliative care referral. Values were measured between fiscal years 2018 to 2020. All values have a 95% confidence interval reported in brackets.

© 2025 Kolla L et al. *JAMA Health Forum*.

eTable 10: Model calibration and AUC between 2016[2017] and 2020[2021]

AUC:

Year of Risk Score Calculation	Area	StdErr	LowerArea	UpperArea
2016	0.8234	0.000463	0.8225	0.8243
2017	0.823	0.000453	0.8221	0.8239
2018	0.8166	0.000471	0.8156	0.8175
2019	0.8276	0.000499	0.8266	0.8286
2020	0.8133	0.000541	0.8122	0.8143

Calibration curve (2016-2020):

© 2025 Kolla L et al. *JAMA Health Forum*.

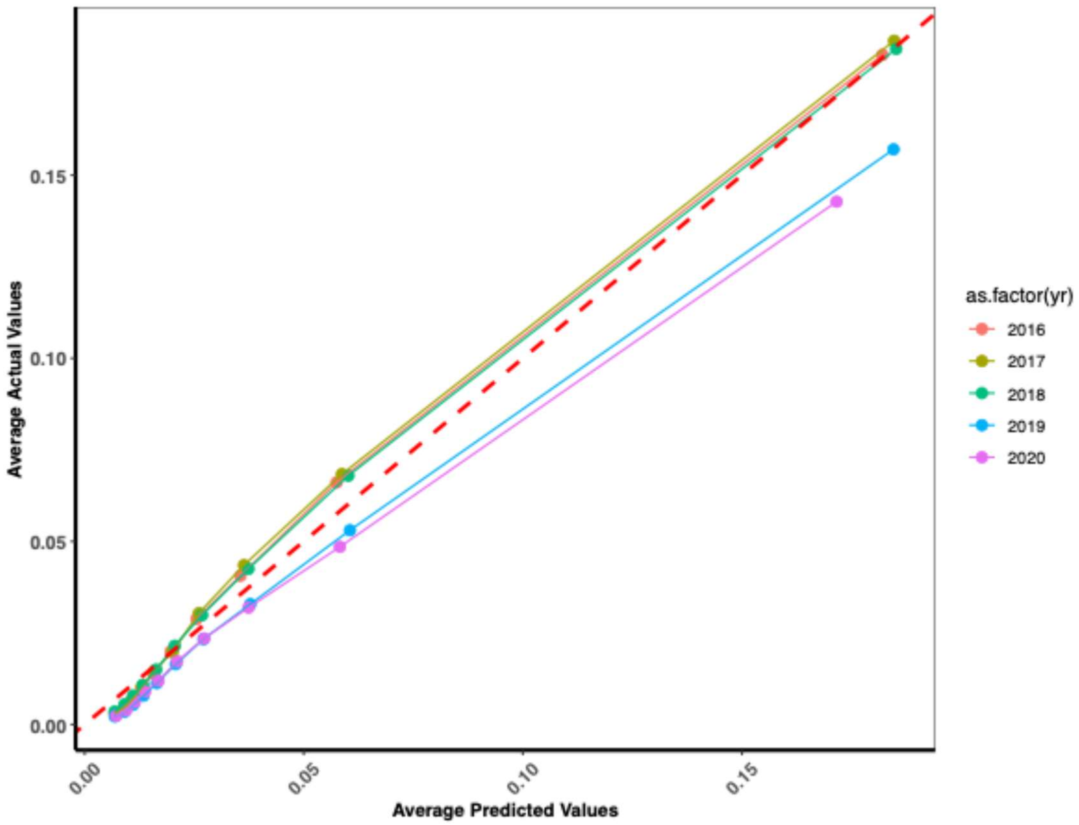

The calibration curve (Figure) highlights the model's alignment between predicted and actual risk across study years. Across the five-year period from 2016 to 2020, AUC remains stable while model calibration worsens in 2019 and 2020, overestimating risk notably at the higher average predicted risk deciles.

eAppendix 1. Algorithm Classification and Performance Metrics

Clinical risk prediction algorithms estimate the likelihood that an individual has a disease (i.e. diagnostic algorithm) or will experience an adverse outcome in the future (i.e. prognostic algorithm). Predictions are numerical values between 0 and 1; and values across patients are summarized in a risk distribution (Figure: Schematic of risk distribution, with distributional tails cut off). Prediction of binary outcomes (e.g. likelihood that a patient experiences an outcome vs. does not experience the outcome) requires a risk threshold to distinguish "high-risk" and "low-risk" patients. Predicted outcomes are compared against observed outcomes to classify how well

© 2025 Kolla L et al. *JAMA Health Forum*.

an algorithm predicted positive and negative cases. These classifications are summarized into confusion matrices (Figure: Confusion Matrix, see below).

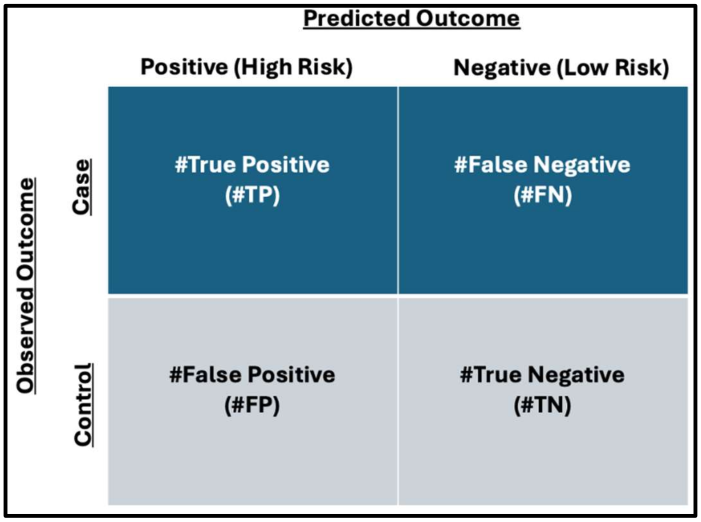

Figure: Confusion Matrix displaying the classification of observations based on observed and predicted outcomes. ‘True Positive' (TP) and 'False Negative' (FN) denote cases where the observed outcome was a case, while 'False Positive' (FP) and 'True Negative' (TN) represent instances where the observed outcome was a control."

© 2025 Kolla L et al. *JAMA Health Forum*.

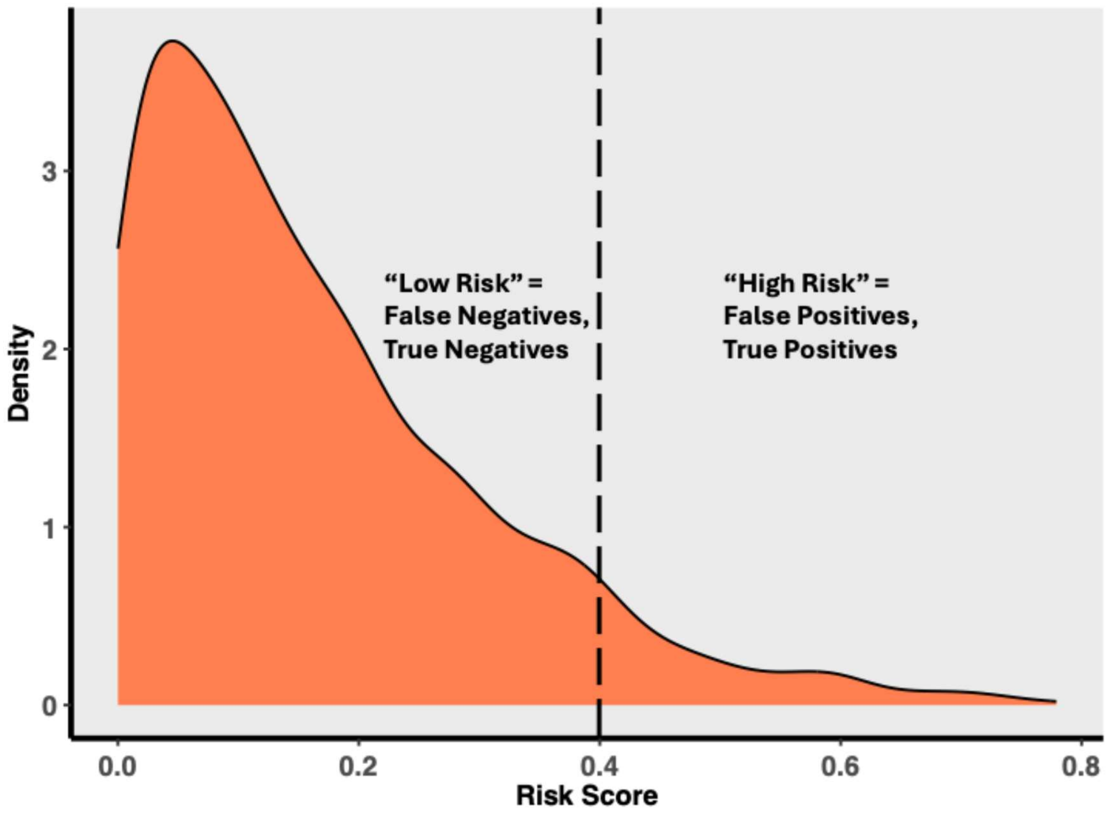

Figure: Schematic of risk distribution, where the dashed vertical line represents risk threshold at 0.4. Scores to the right of the threshold are flagged as “high-risk” and contain values from false positive and true positive individuals. Scores to the left of the threshold are flagged as “low-risk” and contain values from false negative and true negative individuals. Distribution tails are not shown in this illustration.

Risk distributions can be evaluated separately for cases vs. controls (i.e. those who experience an outcome vs. those who do not experience an outcome), as shown below:

© 2025 Kolla L et al. *JAMA Health Forum*.

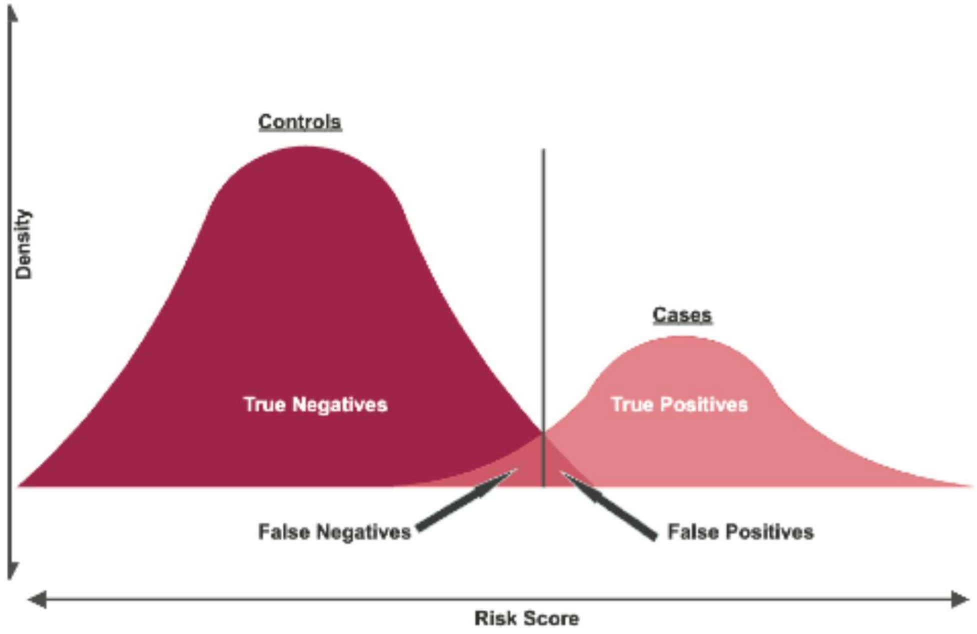

Figure: Distribution of risk scores for control and case groups. The control group (shown on the left) contains True Negatives and False Positives, while the case group (shown on the right) contains True Positives and False Negatives. False Negatives are cases misclassified with lower risk scores, falling within the control distribution, while False Positives are controls misclassified with higher risk scores, overlapping with the case distribution. Distribution tails are not shown in this illustration.

Definitions:

Cases: Individuals who have the disease or experience a future adverse outcome = #TP + #FN

Controls: Individuals who do not have the disease or do not experience a future adverse outcome = #TN + #FP

© 2025 Kolla L et al. *JAMA Health Forum*.

High-Risk: Individuals who are classified by the algorithm as being at great risk for having the disease or for experiencing a future adverse outcome = #TP + #FP

Low-Risk: Individuals who are classified by the algorithm as not being at great risk for having the disease or for experiencing a future adverse outcome = #TN + #FN

True Positive (TP): Case correctly classified as a case

True Negative (TN): Control correctly classified as a control

False Positive (FP): Control misclassified as a case

False Negative (FN): Case misclassified as a control

We measured six clinically relevant algorithm performance metrics (i.e. TPR, FPR, PPV, NPV, Accuracy, and F1 score) in this study. Each metric is a function of algorithm classifications (i.e. TP, TN, FN, FP) and are defined below within the context of prognostic algorithms:

**Metric**	**Calculation**	**Description**
True Positive Rate (TPR)	$\frac{\#TP}{\#TP + \#FN} = \frac{\#\text{correctly identified case}}{\#\text{cases}}$	Proportion of all individuals who experience the outcome in the future (i.e cases)

© 2025 Kolla L et al. *JAMA Health Forum*.

		that are identified as so by the algorithm.
False Positive Rate (FPR)	$\frac{\#FP}{\#FP + \#TN} = \frac{\#\text{incorrectly identified case}}{\#\text{controls}}$	Proportion of all individuals who do not experience the outcome in the future (i.e controls) that are identified as so by the algorithm.
Positive Predictive Value (PPV)	$\frac{\#TP}{\#TP + \#FP} = \frac{\#\text{correctly identified case}}{\#\text{high risk}}$	Proportion of individuals who experience an outcome in the future among all individuals whose predictions exceed a preset risk threshold (i.e. high risk).
Negative Predictive Value (NPV)	$\frac{\#TN}{\#TN + \#FN} = \frac{\#\text{correctly identified control}}{\#\text{low risk}}$	Proportion of individuals who do not experience an outcome in the future among all individuals whose predictions do not exceed a preset risk threshold (i.e. low risk).

© 2025 Kolla L et al. *JAMA Health Forum*.

Accuracy	$\frac{\#TP + \#TN}{\#TP + \#FN + \#FP + \#TN} = \frac{\#\text{correctly identified cases/controls}}{\text{total}}$	Proportion of correctly classified individuals among all individuals.
F1 Score	$\frac{2 * TPR * PPV}{TPR + PPV}$	Function (i.e. harmonic mean) of TPR and PPV. Emphasizes impact of both false negatives and false positives on algorithm performance.

Note: Panel of metrics hold clinical and operational value. For example, true positive rate (TPR) is crucial for understanding the algorithm's ability to identify actual cases, helping clinicians ensure they don't miss patients needing intervention. Positive predictive value (PPV), on the other hand, is key for operational decision-making, as it indicates, on average, how many flagged "high-risk" subjects are actually having a true risk for the outcome, which is vital for optimizing resource allocation and minimizing unnecessary interventions[14].

Pepe, M. S. (2003). *The statistical evaluation of medical tests for classification and prediction*. Oxford university press.

© 2025 Kolla L et al. *JAMA Health Forum*.

eAppendix 2: Risk distribution and dataset shift mechanisms

Algorithms trained on prior patient records can generate predictions for new patients in real-time using routinely collected data at patient visits. One of the main assumptions underlying algorithm development is that the data used to train the algorithm is representative of data that is generated in new testing scenarios (e.g. post-deployment environments). However, changes in clinical practice patterns are characteristic of medicine and can lead to covariate and outcome data distributions that are different from training distributions, a phenomenon known as *dataset shift.* Consequentially, algorithms generate predictions that are less accurate

in environments it is not trained to recognize, leading to decreased performance and inaccurate predictions. Two forms of dataset shift are covariate shift and prior probability shift:

**Type of Dataset Shift ***	**Definition**	**Example**
Covariate Shift	Changes in the distribution of input variables (i.e. covariates)	Case mix of a hospital changes over time (e.g. greater volume of older patients)
Prior Probability Shift	Change in the distribution of the outcome	Prevalence of hospitalization decreases due to reduced access to healthcare facilities during lockdown, shifting the distribution of the outcome to the left over time.

© 2025 Kolla L et al. *JAMA Health Forum*.

*Note: Concept shift, changes in the relationship between input and output variables, was not explored in this study.

Dataset shift mechanisms underlying performance drift can occur in isolation or in unison. Here are three examples of how dataset shifts affect risk distributions and algorithm classifications:

Example 1. Covariate shift, changes in input distribution(s) that lead to a left shift in the predicted risk

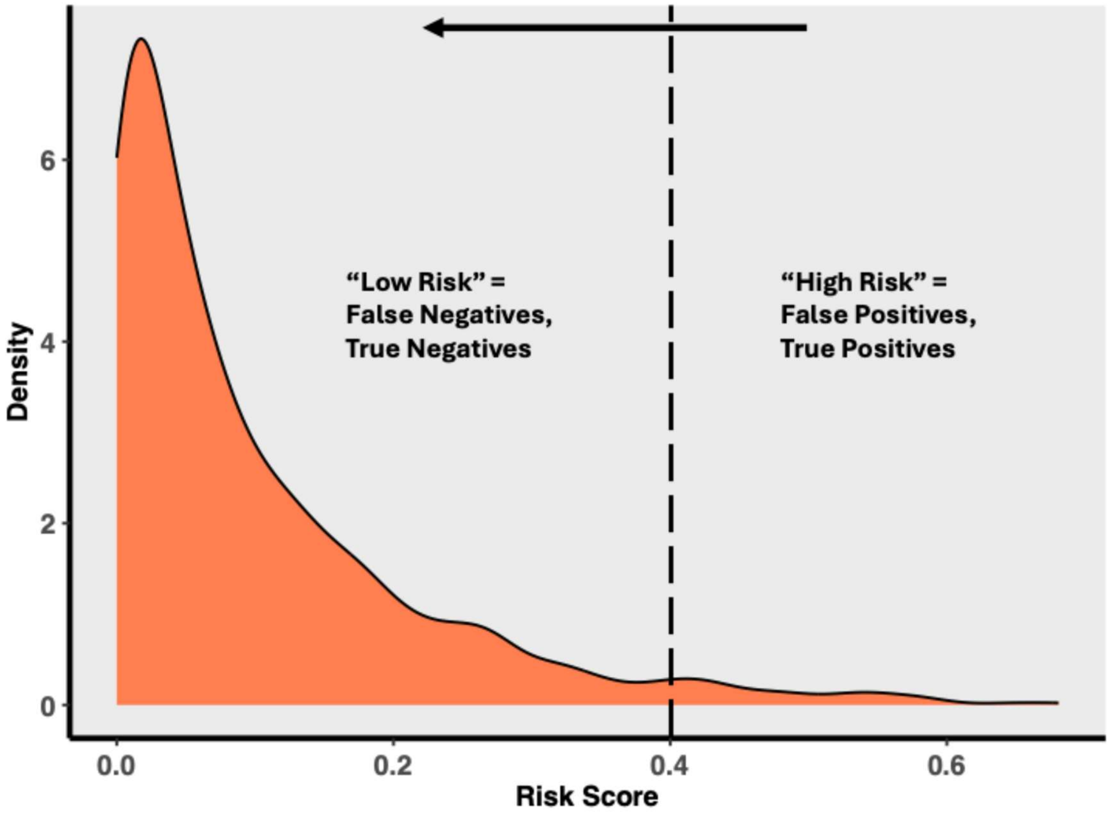

Figure: Impact of covariate shift on the distribution of risk scores, highlighting regions of low and high risk. The dashed line at 0.4 indicates the threshold separating 'Low Risk' (left side) from 'High Risk' (right side), where 'Low Risk' includes False Negatives and True Negatives, and 'High Risk' includes False Positives and True Positives. This distribution can be compared with

© 2025 Kolla L et al. *JAMA Health Forum*.

the risk distribution shown in the 'eAppendix 1--Algorithm Classification and Performance Metrics.'

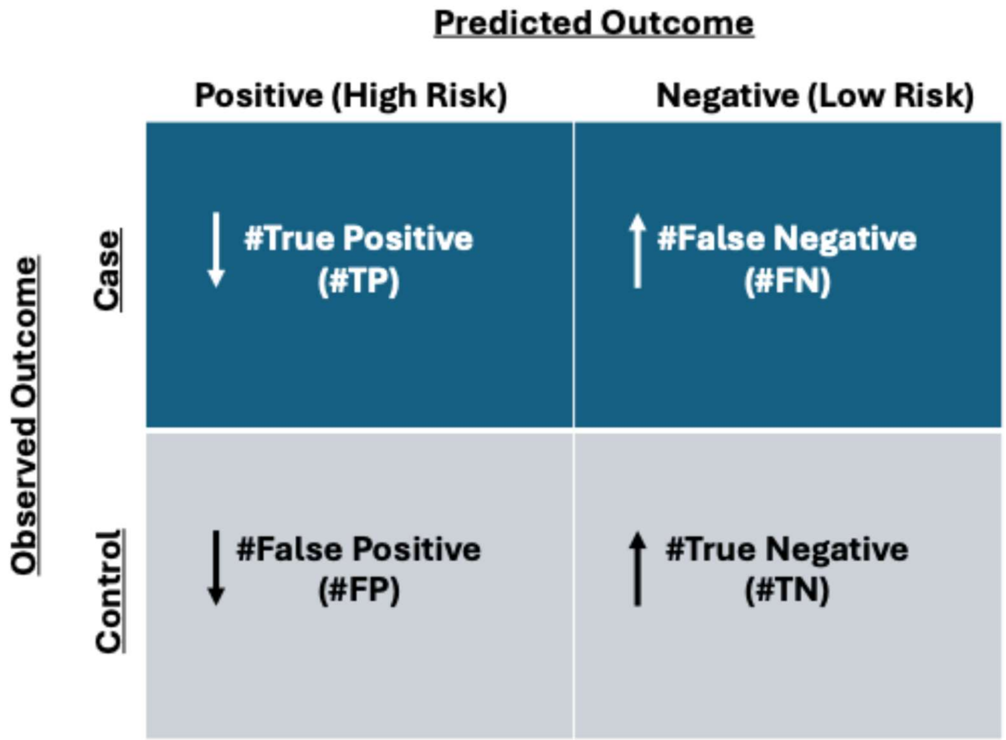

Figure: Impact of leftward covariate shift on algorithm classifications. Holding the number of individuals and risk threshold constant, a uniform leftward shift in the risk distribution would decrease number of high risk individuals, likely decreasing both number of true positives and number of false positives. Concurrently, we would expect an increase in "Low Risk" individuals, shown as an increase of number of false negatives and number of true negatives.

Example 2. Prior probability shift, changes in output distribution (e.g. decreased prevalence of measured outcome)

© 2025 Kolla L et al. *JAMA Health Forum*.

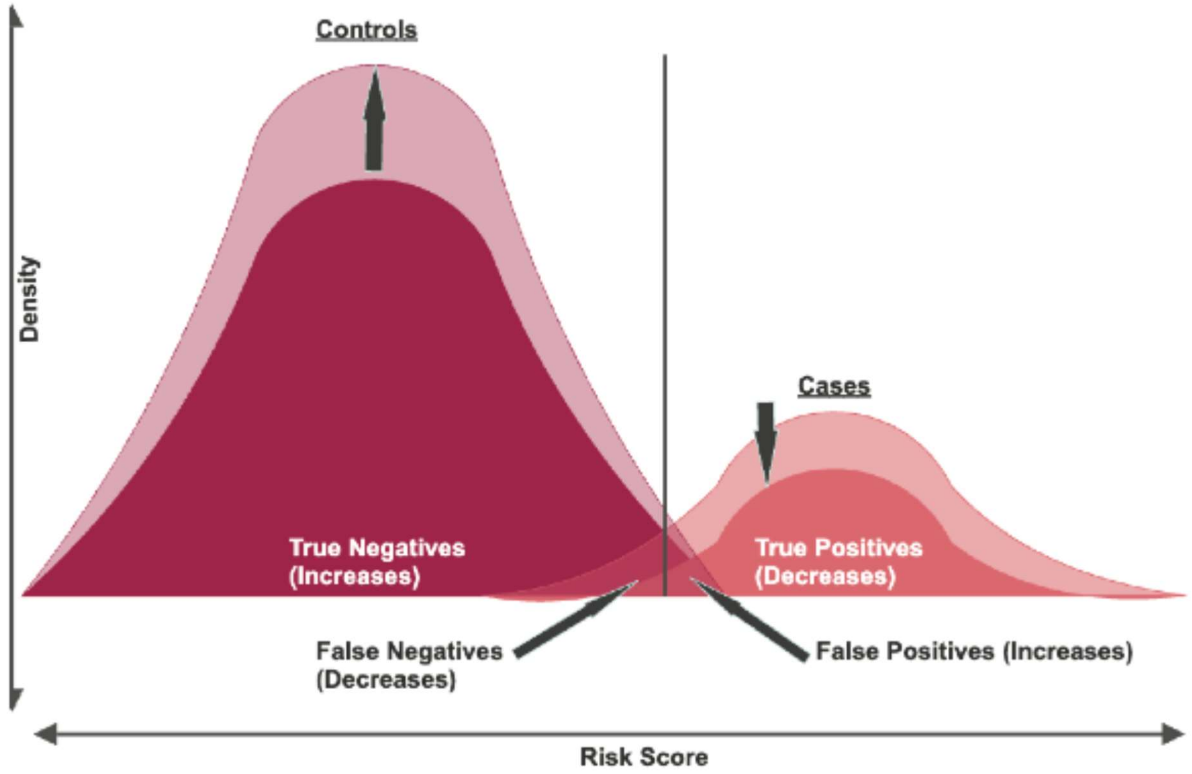

Figure: Impact of decreased prevalence on the risk distributions of cases versus controls. This shift results in an increase in True Negatives and False Positives in the control group, while True Positives decrease in the case group. False Negatives also decrease slightly. This distribution can be compared with the risk distributions for cases versus controls provided in the 'eAppendix 1–Algorithm Classification and Performance Metrics.'

© 2025 Kolla L et al. *JAMA Health Forum*.

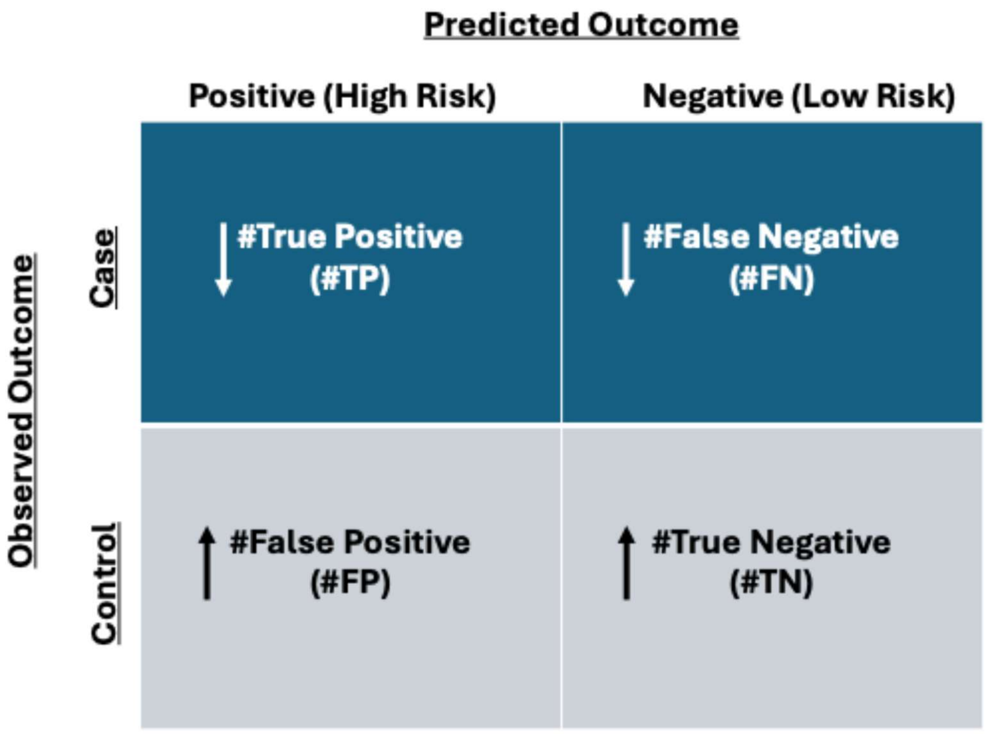

Figure: Impact of decreased prevalence (one form of prior probability shift) on algorithm classifications. Holding the number of individuals and risk threshold constant, decreased outcome prevalence would decrease number of cases in the population and increase number of controls. As a result, we would expect to see greater number of people classified as True Negatives and False Positives; and fewer number of people classified as True Positives and False Negatives.

Example 3. Covariate shift and prior probability shift, concurrent changes in input and output distribution(s)

© 2025 Kolla L et al. *JAMA Health Forum*.

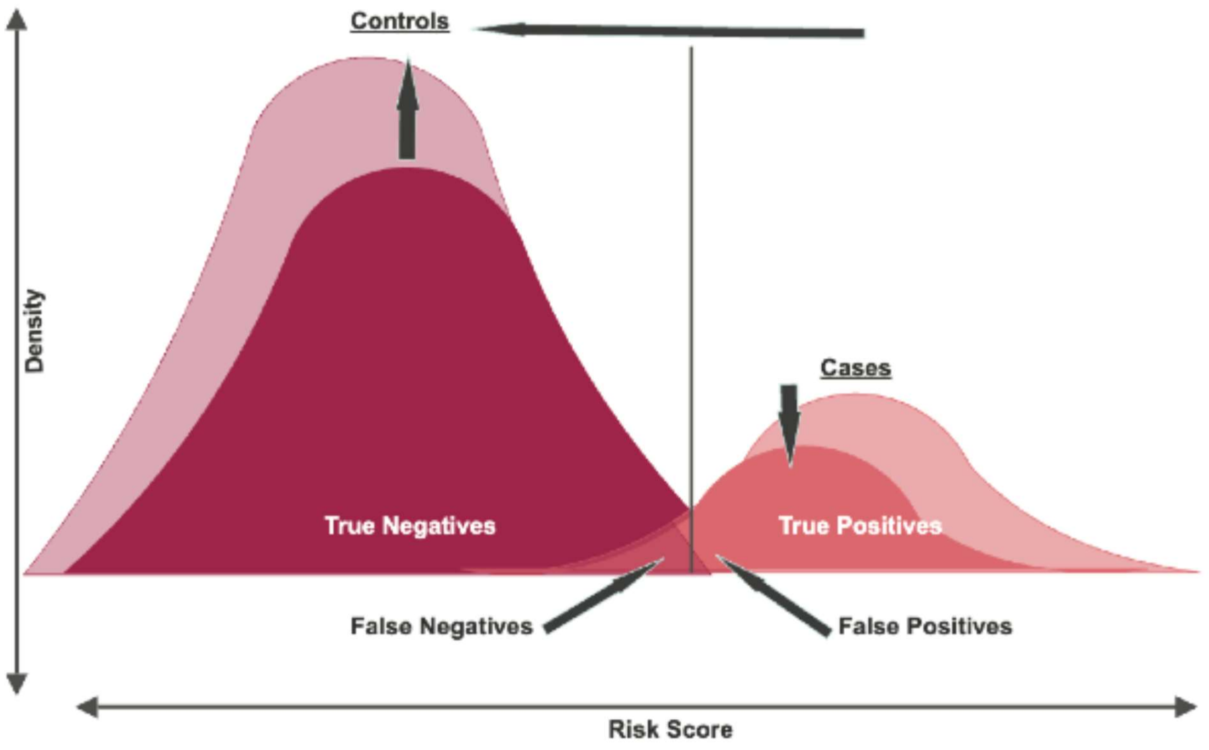

Figure: Impact of decreased prevalence (a form of prior probability shift) and a leftward covariate shift on the risk distributions of cases versus controls. This adjustment increases the density of True Negatives in the control group and decreases the density of True Positives in the case group, while also influencing the balance of False Positives and False Negatives across both distributions.

© 2025 Kolla L et al. *JAMA Health Forum*.

eAppendix 3. Standardized Mean Difference

The standardized mean difference is used to measure the difference between two group means, standardized by their variability, enabling comparisons across variables on different scales. The formula for calculating the standardized mean difference in the proportion of a dichotomous algorithm covariate between the final drift year and the initial drift year is as follows[9]:

$$SMD = \frac{\hat{p_2} - \hat{p_1}}{\sqrt{\frac{\hat{p_2}(1-\hat{p_2})+\hat{p_1}(1-\hat{p_1})}{2}}}$$

,

where $\hat{p_1}$ represents the covariate prevalence in the first year of a drift period and $\hat{p_2}$ represents the prevalence in the final year of a drift period.

15. Austin PC. Balance diagnostics for comparing the distribution of baseline covariates between treatment groups in propensity-score matched samples. *Stat Med*. 2009;28(25):3083-3107. doi:10.1002/sim.3697

© 2025 Kolla L et al. *JAMA Health Forum*.
