# Supplementary material for: Performance Drift in a Nationally Deployed Population Health Risk Algorithm in the US Veterans Health Administration
Source: JAMA Health Forum. 2025 Aug 15;6(8):e252717. doi: 10.1001/jamahealthforum.2025.2717 (PMC12357188; doi:10.1001/jamahealthforum.2025.2717)
Supplement: Supplement 2. — Data Sharing Statement [file jamahealthforum-e252717-s002.pdf]

Data Sharing Statement

Kolla. Performance Drift in a Nationally Deployed Population Health Risk Algorithm in the US Veterans Health Administration. *JAMA Health Forum*. Published August 15, 2025. doi:10.1001/jamahealthforum.2025.2717

Data
Data available: Yes
Data types: Data (not involving human participants), Data dictionary
How to access data: Data to anyone upon approval of a proposal
When available: With publication

Supporting Documents
Document types: Statistical/analytic code
How to access documents: Code to anyone upon approval of a proposal
When available: With publication

Additional Information
Who can access the data: Researchers with an approved proposal
Types of analyses: Analyses consistent with the approved research proposal
Mechanisms of data availability: Data and code are available upon request and approval through the VHA Office or corresponding author
